# Critical assessment of coiled-coil predictions based on protein structure data

**DOI:** 10.1038/s41598-021-91886-w

**Published:** 2021-06-14

**Authors:** Dominic Simm, Klas Hatje, Stephan Waack, Martin Kollmar

**Affiliations:** 1 Group Systems Biology of Motor Proteins, Department of NMR-Based Structural Biology, Max-Planck-Institute for Biophysical Chemistry, Göttingen, Germany; 2 Theoretical Computer Science and Algorithmic Methods, Institute of Computer Science, Georg-August-University Göttingen, Göttingen, Germany; 3 Present Address: Roche Pharmaceutical Research and Early Development, Pharmaceutical Sciences, Roche Innovation Center Basel, F. Hoffmann-La Roche Ltd., Basel, Switzerland

**Keywords:** Computational biology and bioinformatics, Protein function predictions, Protein structure predictions

## Abstract

Coiled-coil regions were among the first protein motifs described structurally and theoretically. The simplicity of the motif promises that coiled-coil regions can be detected with reasonable accuracy and precision in any protein sequence. Here, we re-evaluated the most commonly used coiled-coil prediction tools with respect to the most comprehensive reference data set available, the entire Protein Data Bank, down to each amino acid and its secondary structure. Apart from the 30-fold difference in minimum and maximum number of coiled coils predicted the tools strongly vary in where they predict coiled-coil regions. Accordingly, there is a high number of false predictions and missed, true coiled-coil regions. The evaluation of the binary classification metrics in comparison with naïve coin-flip models and the calculation of the Matthews correlation coefficient, the most reliable performance metric for imbalanced data sets, suggests that the tested tools’ performance is close to random. This implicates that the tools’ predictions have only limited informative value. Coiled-coil predictions are often used to interpret biochemical data and are part of in-silico functional genome annotation. Our results indicate that these predictions should be treated very cautiously and need to be supported and validated by experimental evidence.

## Introduction

Coiled coils consist of two or more α-helices that twist around each other and give rise to a multitude of supercoiled quaternary structures^1,2^. Coiled-coil regions are characterised by hydrophobic residues at the interface between the supercoiled α-helices and by charged and polar amino acids at the outside. This pattern is usually found in heptads (with the amino acids marked as *abcdefg*) where the hydrophobic residues are located in *a* and *d* positions, but slightly different patterns from hendecad and pentadecad repeats are also observed^3–7^. The coordinates of the smallest building block, two closely packing α-helices, can be calculated from parametric equations^8^. This might explain why the coiled-coil dimer was likely the first structural element, for which a sequence—structure—function relationship could be established^9,10^. Accordingly, one of the first tools for predicting protein structure was COILS, which allowed the identification of coiled-coil regions from protein sequences alone^11^. Coiled-coil structures are claimed to be better understood than those of any other fold^12,13^ and are increasingly used as building blocks in the emerging fields of synthetic biology and de novo protein design^14–18^. The most advanced design case so far is likely a coiled coil that can switch between pentameric and hexameric states upon pH-change^19^. Thus, it seems well possible now to design amino acid sequences forming coiled-coil structures with dedicated oligomeric states.

The complementary problem of detecting coiled-coil regions in amino acid sequences is considered to have been solved as well given the deep biochemical understanding of this structural motif. Even if the oligomeric state is not predicted correctly, it is expected that at least the presence and position of the coiled coil is properly recognized. Multiple prediction programs have been developed using different approaches. COILS^11^ and its successor NCOILS (COILS2.2)^20^ match sequences against a fixed length position-specific scoring matrix derived from frequencies at heptad positions. PairCoil^21^ and MultiCoil^22^ expand this concept by adding pairwise residue correlations to the matrix. PairCoil2 is similar to PairCoil but trained with more coiled-coil sequences^23^. Using a different approach, Marcoil calculates posterior probabilities from a windowless hidden Markov model (HMM)^24^. MultiCoil2 is an advancement to MultiCoil and combines the pairwise correlations with a HMM into a Markov Random Field^25^. SOSUIcoil uses a unique concept by discriminating coiled-coil regions from other types of regions applying the canonical discriminant analysis^26^. PCOILS^27^ is an alternative to NCOILS substituting the sequence-profile comparison with a profile-profile comparison. The tools CCHMM^28^ and CCHMM-PROF^29^ also use HMMs, trained with sequences and profiles, respectively. The latest development, DeepCoil, uses a neural network-based method^30^. SpiriCoil does not really predict coiled-coil regions, but scores query sequences against protein profiles of the SUPERFAMILY database, which is thought to represent all proteins of known structure^31^, and passes SUPERFAMILY’s coiled-coil assignments to the new sequence^32^. Some tools can also predict oligomeric states of coiled coils, such as LOGICOIL, PrOCoil, Scorer, and RFCoil, but predicting the oligomeric state is still error prone because of the low number of available protein structures with complex coiled-coil arrangements for training the algorithms^33–36^. In contrast to these many coiled-coil prediction tools, there is a single software, termed SOCKET, that detects knobs-into-holes packing in protein structures^37^.

Given the many available prediction tools, there are multiple studies indicating high sensitivity and specificity in comparative analyses^32,38,39^. The benchmark data used, however, were limited by restriction to a selection of SCOP protein families containing coiled-coil regions (SCOP = Structural Classification of Proteins database)^40,41^, or intersections of SCOP and SOCKET hits. These approaches assessed the sensitivity and specificity against highly restricted data sets and, therefore, strongly overestimated the proportion of true positive coiled-coil predictions. These results do not allow to even estimate the number of false negative cases (no prediction where a coiled coil is present) and false positive predictions (prediction of a coiled coil where there is none) in a representative proteome.

Coiled-coil predictions are part of the standard tool box for in-silico functional genome annotation. In the most extensive comparative study available today, SpiriCoil was used to predict coiled coils in the proteomes of more than 1200 sequenced genomes suggesting that 0.33 to 6.53% of a species’ proteins contain at least one coiled-coil region^32^. A proteome-wide prediction with NCOILS suggested similar proportions^42^. Because most of the proteins predicted to contain coiled coils do not belong to the protein families with known extended coiled-coil regions such as muscle myosin heavy chain and intermediate filament proteins, we wondered how many of these proteins really contain true coiled-coil domains. The most promising approach to evaluate coiled-coil predictions is to compare the predictions with known protein structures. Therefore, we assessed the current status of coiled-coil prediction accuracy by running most of the available coiled-coil prediction tools against all sequences, for which protein structures are known: the entire PDB. Each software was used with default parameters as recommended by the developers and as commonly done in genome annotation pipelines.

## Results

### Prediction of coiled coils in protein structures and their sequences

**Figure 1:**
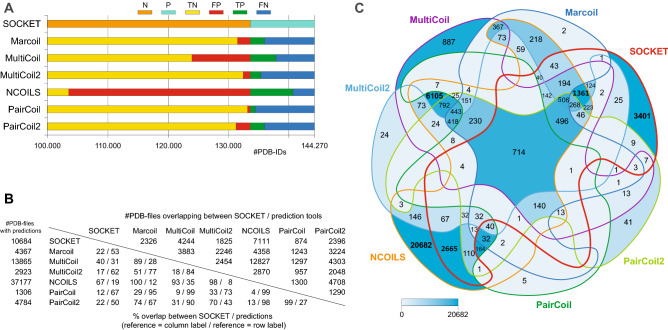
Coiled-coil regions identified by SOCKET and predicted with the respective tools at the level of PDB files. (**A**) Of the 144,270 PDB files 10,684 contain a coiled coil according to SOCKET (P = positives; the remaining 133,586 PDB files represent the negatives = N). Based on the overlap with these SOCKET hits, the coiled-coil predictions of the tools were categorized into four classes: true positives (TP = predictions in same PDB files as SOCKET hits), false positives (FP = predicted coiled coils in PDB files where SOCKET did not detect any), true negatives (TN = no prediction and no SOCKET hit in PDB files), and false negatives (FN = no prediction in PDB file in which SOCKET identified a coiled coil). (**B**) The tools predicted coiled coils in 1307 (PairCoil) to 37,177 PDB files (NCOILS; left column). The numbers in the matrix denote the overlap between SOCKET and every tool, and between any two tools, at the level of hits within the same PDB file (upper part: total numbers; lower triangle: percentage overlap with respect to SOCKET or tool). (**C**) The 7-way Venn diagram shows the subsets of PDB files with SOCKET hits and predicted coiled coils found by the respective combinations of tools colored by number in intersection. The intersection of PDB files with coiled coils predicted by all tools is 714 PDB files, and 1210 PDB files when ignoring PairCoil, the tool with the least predictions.

To create a ground truth to rely on and compare against, we used SOCKET^37^, the de facto standard to detect coiled-coil regions within PDB structures. Instead of using SOCKET data generated by the CC + database^43^, the Periodic Table of Coiled Coils^44^ or the Atlas of Coiled Coils^45^ we generated our own reference to include the latest PDB release possible and for easier integration with the other prediction data generated. The number of coiled coils that SOCKET might miss is expected to be relatively small compared to the size of the PDB. Such cases could be NMR structures with which SOCKET sometimes has trouble dealing with^32^. Within 144,270 PDB files (PDB status 12/2018), SOCKET detected 59,693 components (27,803 coiled coils) in 10,684 (7.4%) PDB files (Fig. 1A; Supplementary Table S1). This means that most PDB files with SOCKET hits contain multiple coiled-coil domains, in different copies of the same biological unit, in different regions of the same protein, or in different proteins if biological units consist of multiple different proteins. From all PDB files we extracted 187,021 unique sequences, meaning that exact copies of the same sequence were removed independent of whether they were present in the same or a different PDB file, while slightly different sequences (e.g. longer N- or C-terminus, insertions) remained. With respect to these unique 187,021 sequences, there are 14,117 (7.5%) distinct coiled-coil sequences (components) as found by SOCKET. This occurrence is similar to that of predicted coiled coils in reported analyses of genome annotations^32^.

To assess the accuracy of coiled-coil predictions from sequences alone, we compared the SOCKET reference set with the results from NCOILS (COILS2.2), PairCoil, PairCoil2, MultiCoil, MultiCoil2, and Marcoil. We did not include SOSUIcoil because it is not accessible anymore. The tools CCHMM and CCHMM-PROF were also excluded. A short test for prediction performance of CCHMM-PROF using the globular and coiled-coil free myosin motor domain resulted in many predicted coiled-coil regions, which are obviously not correct (Supplementary Fig. S1A). CCHMM-PROF requires protein profiles as input. SpiriCoil is also only available via a web interface, caused server errors when used, and was therefore excluded. We also refrained from using the tool PCOILS, the successor of NCOILS, because it runs very slowly and is thus not applicable for the amount of data to be analysed. The latter problem is likely the reason why NCOILS is the tool commonly used in genome annotation projects. In addition, PCOILS was found to be less accurate than Marcoil^38^ and showed the highest overlap with predictions of intrinsically disordered regions^46^. We did not include DeepCoil because running of the software available at the time of performing this analysis (December 2019) was not possible without execution errors. In addition, DeepCoil is limited to sequence lengths of 500 amino acids, which is only slightly larger than the median eukaryotic protein length and much shorter than the length of classical coiled-coil containing proteins such as myosins and kinesins. A test for the prediction performance using a 500 aa region of the myosin-X motor protein^47^ via the DeepCoil web server resulted in mis-prediction of a coiled-coil region at the first IQ motif, which is a calmodulin binding-site, and mis-prediction of a coiled-coil region, where these class-10 myosins contain extended SAH (single α-helix) domains^48^ (Supplementary Fig. 1B). The 500 aa query limit is removed in DeepCoil2 (v. 2.0.1., 30 Nov 2020), but the available (and maybe not completely finished) version of DeepCoil2 does not predict any coiled-coil region in mouse MyoX (Supplementary Fig. 1B; in sharp contrast to DeepCoil “v.1”) and no coiled-coil regions in many of the classical coiled-coil proteins such as the muscle and non-muscle myosin heavy chain proteins (Supplementary Fig. 2). While DeepCoil2 might perform very well on PDB data (it was trained on 90% of all SOCKET hits from the July-2020 version of PDB), DeepCoil2 fails on the tested non-PDB sequences from myosins. In its current status, DeepCoil2 is not a fair competitor in an exclusively PDB-based benchmark study.

To best simulate a common functional protein annotation, we used default parameters for each tool as recommended by the developers, except for setting 21 amino acids as sliding window in all tools for comparability (21 is default in NCOILS).

### Comparing coiled-coil predictions across PDB files

Before investigating the performance of the coiled-coil prediction tools we wanted to get a first glimpse on whether the tools predict coiled coils in the same structures or in different structures. This should result in all possible intersections of reference and prediction tools. Therefore, we analyzed whether coiled coils are found by SOCKET and the prediction tools in sequences of the same PDB file. For this question, only part of a coiled coil (e.g. only one of the sequences in a heterodimeric coiled coil) needs to be identified to classify a PDB file as “coiled coil present”. In addition, this approach ignores whether coiled-coil predictions overlap between tools and the SOCKET reference, and whether sequences contain one or more coiled-coil regions. Accordingly, by this very simplified approach the intersection between reference and predictions is highly overestimated (the tools look better than there results are). In this scenario, there are 10,684 PDB files containing at least one coiled coil found by SOCKET. Surprisingly, the various tools predict coiled coils in strikingly different total numbers of PDB files with PairCoil predicting coiled coils in fewest (1307) and NCOILS in most PDB files (37,177; Fig. 1B and Supplementary Table S1). 33.1% (PairCoil) to 80.9% (NCOILS) of the tools’ predictions were found in PDB files where SOCKET did not find any hit. SOCKET hits are exclusive in 3401 PDB files (31.8% of all SOCKET hits).

Ignoring PairCoil, with which by far the fewest coiled coils were predicted, the minimum overlap of PDB files with coiled-coil predictions from any two tools is 2048 (overlap of MultiCoil2 and PairCoil2 predictions; Fig. 1B). Although this number suggests considerable overlap of predictions in the same PDB files, the opposite is found (Fig. 1C, Supplementary Fig. S3). The predictions overlap by only 11.6% (PairCoil) to 66.6% (NCOILS) with the PDB files containing SOCKET hits (Fig. 1B) indicating that the tools did not predict any coiled-coil regions in the vast majority of the PDB files where SOCKET identified coiled coils. The intersection of PDB files with SOCKET hits and coiled coils predicted by all tools is only 714 PDB files. This number increases to 1210 PDB files if PairCoil, the tool with the fewest predictions, is ignored. All tools predicted coiled coils in 230 PDB files (648 when ignoring PairCoil) where SOCKET did not identify any, potentially indicating structures outside SOCKET’s default cut-off and structures difficult to resolve by SOCKET (e.g. some NMR structures). 9399 PDB files contained coiled coils predicted by at least two tools but did not contain SOCKET hits. This number is considerably higher than that of SOCKET hits overlapping with at least one of the tools (7283 PDB files). While almost all PairCoil and Marcoil predictions overlapped with SOCKET hits or predictions of at least one other tool, PairCoil2, MultiCoil, MultiCoil2, and NCOILS exclusively predicted coiled-coil regions in 41 (0.9% of PairCoil2 predictions), 887 (6.4%), 24 (0.8%), and 20,682 (55.6%) PDB files, respectively (Fig. 1C). Irrespective of the individual performance of each coiled-coil prediction tool, these intersection data demonstrate that the tools predict coiled-coils in very different sequences.

### No effect of sequence redundancy on binary classification metrics

**Figure 2:**
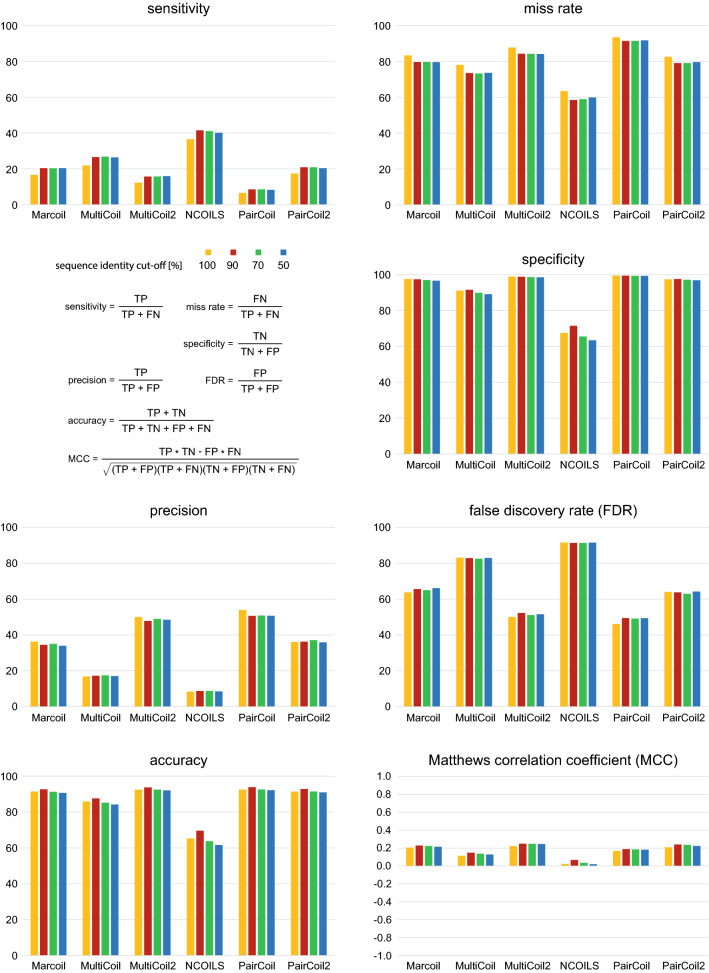
Performance of coiled-coil prediction tools in dependence of PDB sequence redundancy. The performance was analysed for four data sets with decreasing levels of sequence redundancy. With respect to these data sets, the sequences containing a coiled coil according to SOCKET are classified as positives while the remaining sequences represent the negatives. Based on the minimum requirement for overlap with these SOCKET hits (a single amino acid overlap), the coiled-coil predictions of the tools were categorized into four classes: true positives (TP = predictions overlap SOCKET hits), false positives (FP = predicted coiled coils do not overlap SOCKET hits), true negatives (TN = no prediction and no SOCKET hit in sequence), and false negatives (FN = no prediction in sequence region where SOCKET identified a coiled coil). The plots show the performance of the coiled-coil prediction tools based on commonly used statistical measures. A Matthews correlation coefficient (MCC) of + 1 indicates a perfect prediction, predictions with MCCs around 0 are no better than random, and a MCC of − 1 represents total disagreement between prediction and reference.

To evaluate the performance of the coiled-coil prediction tools, we analysed their overlap with SOCKET hits. Of the 187,021 unique sequences in the PDB files, 14,117 contain SOCKET hits, which are defined as positives here. For simplicity, we required a single amino acid overlap between SOCKET hit and coiled-coil prediction for the prediction to be classified as “true positive”. Redundancy in the data is only a problem, if it does not apply to all binary classification categories similarly. Therefore, every filter based on a user-defined criterion, for example selecting proteins whose SCOP family contains coiled-coils, excluding all-beta-strand proteins, or preferentially selecting coiled-coil containing proteins from clusters of otherwise similar proteins, would introduce a bias on the data set taking effect on only the positives or the negatives. Previous comparative analyses used such filters and it has not been investigated whether these filters influenced the performance metrics (Supplementary Notes). To exclude that sequence redundancy in the PDB influences coiled-coil tool evaluation we reduced the redundancy of the 187,021 unique sequences with CD-HIT^49^ applying 90%, 70% and 50% sequence identity cut-offs resulting in 49,311, 39,394 and 30,397 unique sequences, respectively. The drastic reduction by 74% in sequence space from no redundancy to 90% sequence identity nevertheless did not result in considerable changes in the performance of the binary classification (Fig. 2, Supplementary Table S2). The sensitivity of the prediction tools increased by two to five percent and the corresponding miss rates decreased by the same numbers. All other metrics are almost identical. Most notably, there is no change in any of the performance metrics if the sequence redundancy is further decreased from the 90% to 70% and 50% sequence identity. Although values over 90% for several metrics such as specificity and accuracy indicate strong performance of the coiled-coil prediction tools (except for NCOILS), a close look at other metrics demonstrates that the overall performance is instead close to random and might even strongly misguide interpretation of bioinformatics analyses and biological experiments.

### Performance of the coiled-coil prediction tools

All described performance metrics measure the classification quality of either of true and false positives and negatives, and all rely on the characteristics of the data set, i.e. the proportion of positives and negatives. Because the benchmark data set is the entire PDB and all its unique sequences, the number of negatives is much higher than the number of positives (6.2% positives at 90% sequence identity, 7.6% at 100% identity). Accordingly, the specificity and accuracy of the tools are very high, while sensitivity and precision are rather low. It is obvious that the performance of the tools cannot be evaluated just based on these metrics. In case of imbalanced data, the Matthews Correlation Coefficient (MCC) represents the best overall measure to evaluate the performance of binary classifiers. The MCC score ranges from − 1 (totally wrong classification, or perfect classification of the opposite) to 0 (random classification) to + 1 (perfect classification)^50^. Here, requiring overlap of only a single amino acid between SOCKET hit and coiled-coil prediction, the MCC indicates random prediction in case of NCOILS (MCC of 0.02) and close to random prediction for all other tools (MCC of 0.22 for MultiCoil2 being the highest value; Fig. 2). It is important to note that also the MCCs are independent of sequence redundancy reduction.

While random prediction, by wording, suggests a flipped coin chance to have a coiled coil in a sequence when tools predict one, there are two other metrics very important for the experimental biologist. The false discovery rates (FDR), which denote the percentage of false predictions compared to all predictions, show that the actual percentage of false predictions is considerably higher than random, with 83% for MultiCoil and 91% for NCOILS (Fig. 2). For the other coiled-coil prediction tools, the chance of having predicted a true coiled coil is slightly better than flipping a coin (precision of 36–54%, Fig. 2). For the experimental biologist this means that the chance is higher that a coiled coil predicted with one of the many available NCOILS web server is in fact not a coiled coil than the chance that the predicted coiled coil might really be present in the protein. The other important metric is the miss rate, which denotes the percentage of elements in the reference data that were not predicted. The analysis shows that the prediction tools missed from 59 to 63% (NCOILS; range across the redundancy reduced data sets) to 92–93% (PairCoil) of the SOCKET reference coiled coils. For the experimental biologist this means that chances are considerably higher than flipping a coin that a coiled coil is present in a sequence of interest where the prediction tools did not predict any.

### Performance of the coiled-coil prediction tools compared to naïve models

**Figure 3:**
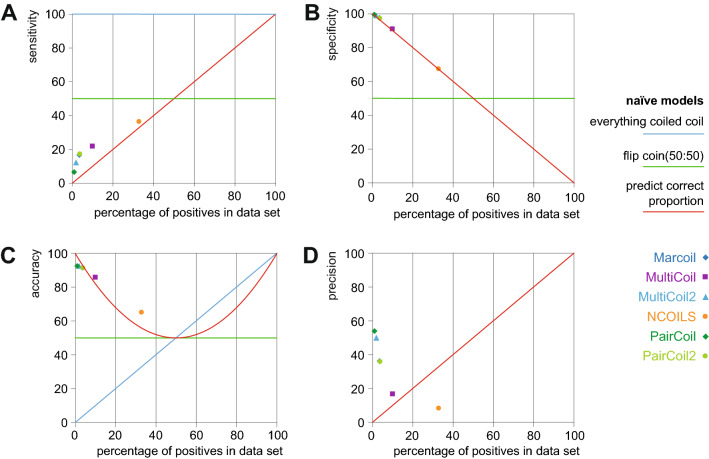
Performance of coiled-coil prediction tools compared to three naïve models.The plots present statistical measures based on three naïve models as described in the legend. The respective performance of the coiled-coil prediction tools based on the unreduced data is plotted for comparison.

Because values over 90% for some of the performance metrics such as specificity and accuracy indicate good performance we compared these with the results of three naïve classification models (Fig. 3). First, we calculated the same metrics assuming that all sequence is classified as coiled coil. Second, we used a coin flip model where half of the cases are coiled coils and the others are not. Third, we assumed that we know the proportion of true coiled coils in the data and randomly predict coiled coils with the same proportion. While the first model assumes an extreme case and the second would provide a reasonable baseline for a balanced data set, the third model provides a good baseline for the coiled-coil prediction tools. Therefore, the base level to estimate the performance of each prediction tool would be the performance obtained if the identical proportion were predicted randomly. Compared to this naïve model, the sensitivity of all prediction tools is slightly, but not considerably better (Fig. 3A). The specificity is almost exactly identical to the specificity obtained if the same number of coiled coils were predicted randomly (Fig. 3B). The accuracy of Marcoil, MultiCoil2, PairCoil and PairCoil2 is lower than in the naïve model, while that of MultiCoil and NCOILS is slightly higher (Fig. 3C). The precision of the tools is highest for the tools with the lowest number of predictions (Fig. 3D). The precision of all tools is considerably better than the naïve model, except for NCOILS, which is considerably worse. From all these metrics it is clear that the coiled-coil prediction tools do not significantly outperform the naïve model of predicting the same proportion randomly.

### Effect of a length cut-off filter on coiled-coil prediction performance

Because SOCKET might identify considerably more short coiled coils than prediction tools do, two sequence length cut-offs were applied and reference SOCKET hits and coiled-coil predictions shorter than 14 amino acids and 21 amino acids were excluded from each data set (Supplementary Fig. S4, Supplementary Table S2). As discussed above, this is not a scientific decision based on data that show that short coiled coils do not exist but a subjective decision based on the observation that coiled-coil prediction tools perform bad in predicting these types of coiled coils. With respect to reducing redundancy of the PDB sequence space the two cut-offs don’t have any effect. At each cut-off, the tools show the same performance for the 100, 90, 70 and 50% sequence identity data sets (Supplementary Fig. S4), indicating again that reducing redundancy is not necessary for evaluating benchmark studies based on PDB sequence data. However, the prediction tools show increasing performance when applying the 14 and then the 21 amino acid cut-offs compared to no length cut-off (Supplementary Fig. S4). The sensitivities increase considerably, and this is the main reason for the increased MCCs, although the values are below 0.4 for all tools and all cut-offs. The increase in sensitivity is a direct consequence of the cut-offs excluding the short coiled coils that the tools rarely predict. NCOILS highly overpredicts coiled coils, and therefore shows the highest specificity and lowest miss rate. The miss rates for the other tools are still above 50% in case of the 14 amino acid cut-off, and decrease to 34% (MultiCoil) to 71% (PairCoil) for the 21 amino acid cut-off (Supplementary Fig. S4). The increase in sensitivity comes to the cost of precision, which decreases by 5–17%. Accordingly, the false discovery rates increase by the same numbers. This analysis demonstrates that sequence redundancy in the PDB does not matter, but, of course, applying a cut-off or filter on the positives does. The results show the performance of the tools with respect to predicting long coiled-coils, and not with respect to predicting coiled coils in general.

### Extent of structural overlap between coiled-coil predictions and SOCKET hits

In contrast to simple absence/presence counting, evaluation of overlap is getting more difficult the shorter the regions and peptide segments are and the more complex the overlapping patterns become. Overlap patterns such as length and/or contiguity of the predictions depend on prediction tools and parameters. Therefore, considerable care must be taken that the scoring scheme for evaluation does not prefer one over the other pattern. For example, the muscle myosin heavy chain proteins are well known to assemble into homo-dimers based on their long, coiled coil forming tail domains. Coiled-coil predictions on a human adult skeletal muscle myosin heavy chain protein sequence result in different sets of coiled-coil regions with different start and end positions, different switches in the predicted heptad registers, and different predictions of oligomerisation state from dimer and trimer to tetramer (Supplementary Fig. S5). It is obvious that predictions of long, uninterrupted coiled-coil regions should be preferred, and that breaks in these very extended coiled-coil regions might be attributed to over- and underwinding of the twisted α-helices. This is very different in the opposite case, for example α-actinin, which forms intra-sequence coiled coils by folding into helix-loop-helix segments. There, the prediction of interrupted coiled coils would be highly preferred over single long, uninterrupted coiled coils. Coiled-coil prediction tools cannot distinguish between intra-sequence and inter-sequence coiled coils.

A method to evaluate very short patterns is the segment overlap score (SOV) that has been developed to compare known and predicted secondary structural elements^51–53^. In contrast to a per-residue score, which judges the percentage of individual overlapping positions, the SOV positively weights contiguous longer overlapping segments and reduces the influence of considered less significant features such as slightly different segment lengths and/or positions. In the current version from 1999^52^, contiguous segments are rated higher than multiple shorter segments when compared to a long reference segment even if the total number of positions is considerably lower, compared to the initial version from 1994^51^. However, it is not clear why there is a strong difference in weighting between the case where the reference consists of multiple segments and the prediction is contiguous and the case where the reference is contiguous and the predictions are multiple segments, why additional splits in overlapping segments do not contribute linearly to down weighting (and why they contribute differently with respect to a contiguous reference or prediction), and why false positives of different features (e.g. α-helix versus β-strand) are less disfavoured than false positives of no feature. Because coiled coils are intermediate between single large features and (potentially) multiple short segments, we decided to apply a percentage overlap to each coiled coil. This approach slightly favors the coiled coil predictions, which are usually longer than the reference because of the window-based prediction algorithms, and does not average coiled-coil evaluations in case sequences contain multiple reference coiled coils and/or predictions.

**Figure 4:**
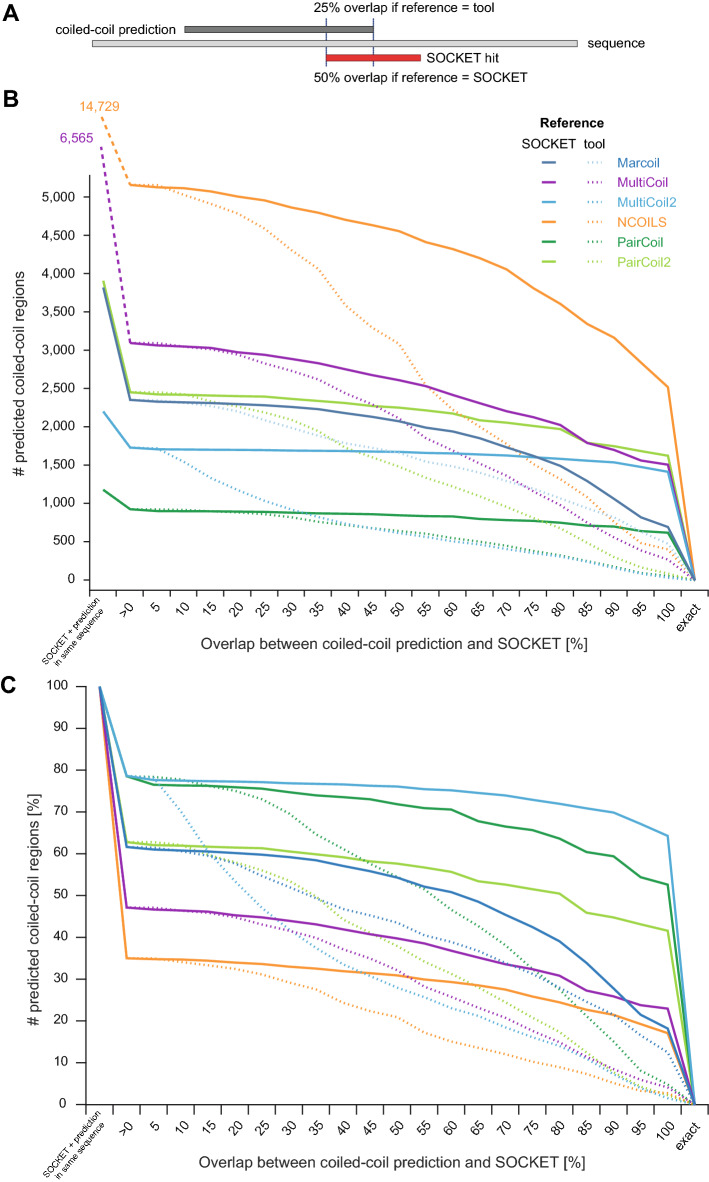
Overlap of coiled-coil predictions with SOCKET hit regions. (**A**) Schematic drawing of a coiled-coil prediction overlapping a SOCKET hit. The ratio of overlap between prediction and SOCKET hit is different depending on whether the prediction or the SOCKET hit is taken as reference. (**B**) The plot shows the total number of coiled-coil predictions in dependence of the degree of overlap with the SOCKET hits. The values at the left side indicate the number of PDB sequences containing both a SOCKET hit and a coiled-coil prediction. For each tool, the number of overlapping hits is counted once with taking the coiled-coil prediction as reference (solid lines) and once with the SOCKET hits as reference (dashed lines). (**C**) This plot is similar to (**B**) but shows the percentage of overlapping regions with respect to the overlap ratio. The number of sequences containing both a coiled-coil prediction and a SOCKET hit is set to 100% for each tool. A similar plot with the number of predictions overlapping a SOCKET hit with at least a single amino acid set to 100% is shown in Supplementary Fig. S4.

Considering possible tool-dependent bias in determining start and end positions of the predictions we determined the number of predictions overlapping SOCKET hits in dependence of reference (either SOCKET or tool) and degree of overlap (Fig. 4, Supplementary Fig. S6, and Supplementary Table S3). At least, each predicted coiled-coil sequence should overlap with a single amino acid of a SOCKET region. At that minimum level only 19,036 (26.7%) of the 71,393 coiled-coil regions predicted by NCOILS and found by SOCKET in the same PDB files overlap. This indicates that the majority of the NCOILS predicted coiled coils do not overlap with SOCKET hits, although SOCKET hits and predicted coiled coils were found in the same PDB file. The percentage of overlapping regions (single amino acid criterion) is highest for MultiCoil2 and SOCKET hits (5590 of 8346 regions, 67.0%; Fig. 4 and Supplementary Fig. S6). Taking the prediction tools as reference and requiring overlap of at least 50% of their predicted sequence regions with SOCKET hit regions, only 13.4% (MultiCoil) to 34.0% (PairCoil) of the tools’ predictions match this criterion. When requiring at least 80% overlap between predicted coiled-coil and SOCKET hit regions, the fraction of overlapping regions decreases further to 5.4% (NCOILS) to 17.5% (PairCoil). When taking SOCKET hits as reference, 23.6% (NCOILS) to 65.0% (MultiCoil2) of the predictions overlap with at least 50% of respective SOCKET hit regions. The percentages of overlapped SOCKET hit regions only slightly decrease with increasing size of overlap when using SOCKET hits as reference. This shows that predicted coiled-coil regions are in general considerably longer than SOCKET hit regions and therefore reach into protein structural regions without knobs-into-holes packing indicative of coiled-coil helix-helix interactions.

### Requiring more overlap between reference and prediction decreases prediction performance

In the performance analyses shown so far, only a single amino acid overlap was required. By this minimum requirement, many predictions matching unrelated structural regions were also considered true positives although they are, by inspecting the structures, in fact false positives. Because SOCKET hits are usually shorter than coiled-coil predictions, using the SOCKET hits as reference for the comparison of the overlap will result in better performance metrics for the prediction tools. Accordingly, we increased the required overlap in steps of 5% for all data sets described before, the four data sets with decreased sequence redundancy and the data sets with additional coiled-coil length cut-off. With respect to reducing redundancy of the PDB sequence space changing the required overlap shows a marginally effect on the performance metrics (Supplementary Figs. S7 and S8; Supplementary Table S4). At each overlap proportion, the tools show almost the same performance for the 100, 90, 70 and 50% sequence identity data sets. However, the performance decreases when increasing the required overlap (Supplementary Fig. S8).

### Patterns of coiled-coil predictions

**Figure 5:**
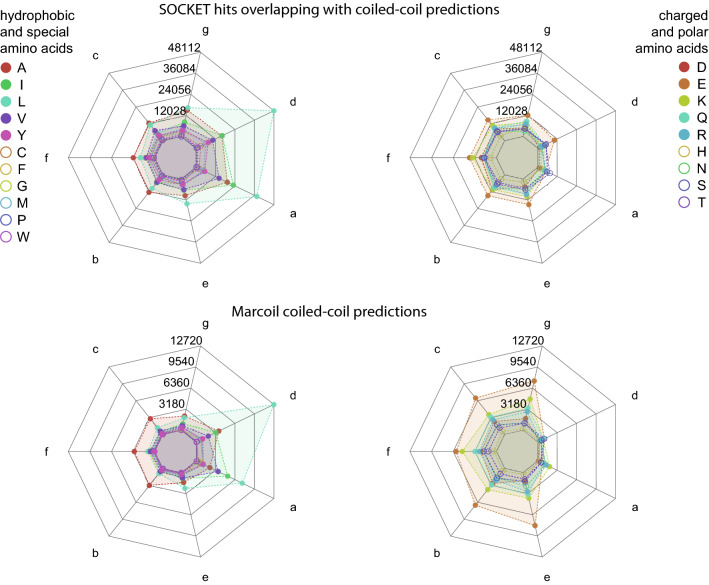
Amino acid preferences at heptad positions *abcdefg*. The spider plots show the distribution of amino acids at each heptad position. Hydrophobic and special amino acids are shown on the left and hydrophobic and polar amino acids on the right for better orientation. SOCKET not only detects “classical” coiled coils but interacting α-helices within globular protein structures. To reveal possible differences in the amino acid distributions among these two groups, SOCKET hits overlapping (shown here) and not overlapping (Supplementary Fig. S4) coiled-coil predictions were analysed separately. The distribution of hydrophobic and charged amino acids is slightly less biased in the latter structures. Marcoil predictions show strong bias for leucine and isoleucine at the interior positions *a* and *d*, and for glutamate at all other positions. The heptad patterns of the other predicted coiled coils show similar distributions. The letters at the axes denote the heptad register positions. Data values at grid lines refer to amino acid counts at each heptad position over all heptads. Amino acids with high proportions are shown as filled circles and amino acids with low proportions as unfilled circles.

The heptad pattern characteristic for almost all coiled-coil regions is found in all SOCKET regions (Fig. 5 and Supplementary Fig. S9). There is a clear preference for leucines in *d* positions compared to *a* positions, and increased propensity for isoleucines and valines in *a* positions compared to *d* positions. There is strong discrimination against glutamates in *a* positions and lysines and arginines in both *a* and *d* positions. However, there are significantly more leucines in *e* and *g* positions than in *b*, *c*, and *f* positions, and there is no discrimination against glutamates in *d* positions (Fig. 5). SOCKET might also detect knobs-into-holes packed α-helices buried within e.g. globular structures, which might not be detected by coiled-coil prediction tools. However, the amino acid distributions at heptad positions were almost identical between SOCKET hits, which overlap regions where tools also predict coiled coils, and SOCKET hits where tools did not identify any (Fig. 5 and Supplementary Fig. S9). The strongest difference between the two patterns is the slightly lower preference for leucines and less discrimination against charged residues. This comparison indicates that from the perspective of amino acid distribution at heptad positions the SOCKET-determined coiled coils in the 3401 PDB files, where the tools did not predict any coiled coil, are not very different from the SOCKET hits overlapping coiled-coil predictions. The patterns of the predicted coiled-coil regions are very similar with respect to each other and to the SOCKET pattern although more discriminating against glutamates and lysines in *a* and *d* positions (Fig. 5).

**Figure 6:**
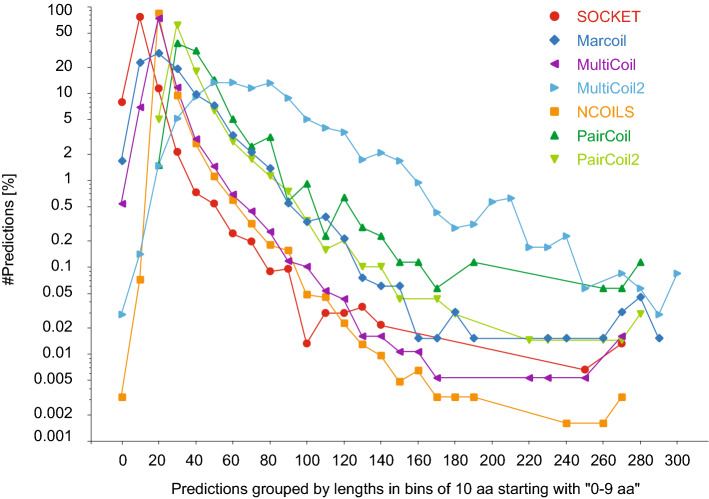
Length of SOCKET hits and coiled-coil predictions. SOCKET hits and coiled-coil predictions were grouped by length in bins of ten amino acids, and the number of hits/predictions in each bin plotted in percent with respect to the total number of hits/predictions of the respective tool.

The different curve shapes for SOCKET hits overlapping predictions and predictions overlapping SOCKET hits (Fig. 4) already showed that coiled-coil predictions are longer regions than SOCKET hits. This is supported by the length distributions of the coiled-coil regions (Fig. 6). Most SOCKET hits are 10 to 19 amino acids long, while most Marcoil, MultiCoil and NCOILS regions are 20 to 29 amino acids long, and most PairCoil and PairCoil2 regions are 30 to 39 residues long. MultiCoil2 regions show a very different length distribution with no specific preference for a certain length. Instead, MultiCoil2 seems to combine consecutive helices, e.g. the repeat regions of helical bundle forming proteins such as spectrin and α-actinin, into single super-long coiled-coil regions.

### Coiled-coil predictions map to all types of secondary structural elements

**Figure 7:**
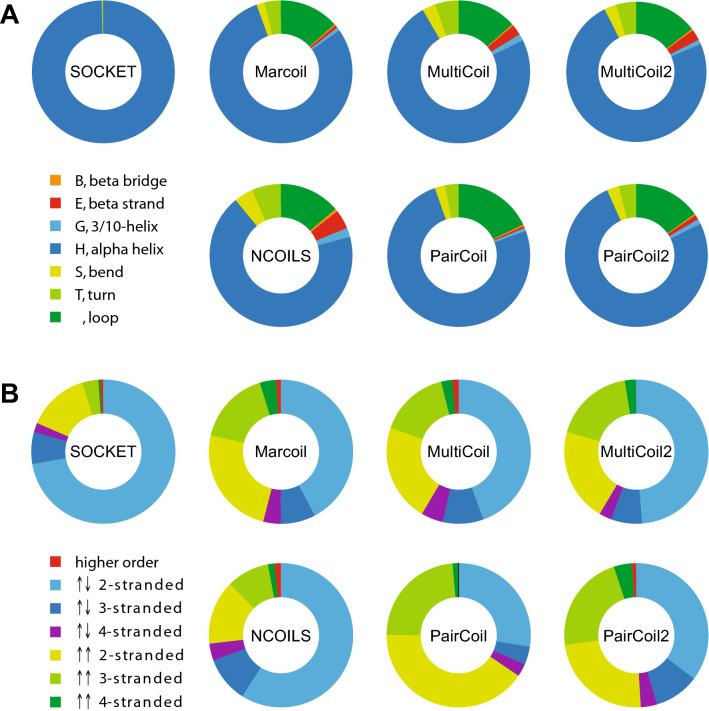
SOCKET hits and coiled-coil predictions matching protein structures. (**A**) The plots represent the matching of all SOCKET hits and all coiled-coil predictions with secondary structure elements as determined by DSSP. The secondary structure assignment for each amino acid was read from the DSSP output, the assignments summed up for each element, and the distribution of elements determined for each tool in percent. (**B**) Coiled-coil predictions on sequence alone are not biased for certain oligomeric states. As reference, the distribution of oligomeric states as determined by SOCKET is shown, with parallel and antiparallel arrangement of the α-helices separated. For each coiled-coil prediction tool, only those predictions were selected that overlap at least 50% of a SOCKET hit, and the oligomeric state assignments of the respective matched hits were collected.

The considerably deviating matchings of SOCKET-determined coiled-coil regions and predicted coiled coils with respect to the PDB structures prompted us to look at the secondary structural elements of matched regions. As ground truth for the secondary structure we relied on DSSP (Define Secondary Structure of Proteins)^54,55^. DSSP assigns secondary structure elements to amino acids based on hydrogen-bonded and geometrical features extracted from X-ray coordinates and is the standard tool at the Protein Data Bank (RCSB PDB) for assigning secondary structures. As expected by SOCKET’s algorithm, which selects α-helical regions assigned by DSSP and uses these to detect knobs-into-holes packing in the protein structures, 99.994% of the amino acids within SOCKET hits match to α-helical regions (H in DSSP notation) while the remaining 0.006% match to loops (“blank “; Fig. 7A and Supplementary Table S5). In contrast, 20.8% (Marcoil) to 31.5% (NCOILS) of the regions predicted to be coiled coils do not fall into α-helices, and considerable parts of MultiCoil (2.6%), MultiCoil2 (2.6%), and NCOILS (5.0%) predictions match to β-strands (Fig. 7A and Supplementary Table S5). To allow visual inspection of these rather surprising results and detection of potential mis-assignments or systematic deviations we implemented a web-interface to the analysis database providing a search interface, a structure viewer, and sequence-based representations of all predictions in comparison. The web-interface can freely be accessed at https://waggawagga.motorprotein.de/pdbccviewer.

### Parallel and antiparallel coiled coils, and oligomeric states

Coiled coils can have parallel and antiparallel arrangements of the α-helices and take part in many different oligomeric assemblies^44^. The “classical” coiled coil is a parallel homodimer, and accordingly the protein training data of the first prediction tools consisted of parallel homo- and heterodimers such as myosins, tropomyosins, kinesins, and intermediate filament proteins. In the PDB by far the most detected arrangement is the antiparallel 2-stranded coiled coil (72.1%) followed by the parallel 2-stranded coiled coil (14.1%; Fig. 7B). NCOILS’ predictions have the largest overlap with SOCKET hits and thus the most similar distribution of arrangements with SOCKET. PairCoil shows the strongest bias of all prediction tools for detecting parallel 2-stranded coiled coils. Marcoil, MultiCoil, MultiCoil2, and PairCoil2 all have similar distributions with bias towards parallel 2-stranded and 3-stranded coiled coils (Fig. 7B). These data show that the prediction algorithms do not exclude certain arrangements, be it the direction or the number of involved α-helices.

### The polyglutamine puzzle

It has been suggested that (Q/N)-rich prions and polyQ-expanded proteins form coiled-coil structures based on the polyQ and neighbouring regions^56^. There are 23 structures now in the PDB containing stretches of at least six glutamines (Table 1). In only one of these structures, 3PJS, the polyQ region is the α-helical extension of a tetrameric coiled-coil structure, while in the others these regions do not take part in any oligomerisation. In 3PJS, the polyQ region is part of an α-helix, but this region does not interact with any neighbouring region. The polyQ region is therefore not part of a coiled coil but part of a single α-helix (SAH). In addition, the polyQ region is not present in any natural sequence of this protein class but the result of multiple mutations to facilitate structural and biochemical analyses. In five structures, the polyQ region is the C-terminal end and extends as SAH domain into the solvent. In the remaining structures, the first two to four glutamines succeeding an extended α-helix often extend this α-helix while the remaining glutamines are mostly flexible and not visible in the crystal structures. Thus, there is no structural support for polyQ-regions forming coiled coils yet.

**Table 1:** Crystal structures of proteins containing stretches of at least six glutamines, aspartates or glutamates.

PDB id	Poly“X” stretch	Observed structure
1U6F	QLQQLQ_6_	Turn, bend
2DMS	Q_7_	Turn, bend
2NB1, 4A9Z	Q_6_HQ	Helical, SAH
2OTU, 2OTW	Q_10_G	Turn, bend
1QB3	Q_16_HQTQ	No structure
3IO4, 3IO6, 3IOR, 3IOT, 3IOU, 3IOV, 3IOW	Q_17_	Multiple conformations, mainly turn and bend, partially helical
3PJS	QEQ_6_	Helical, SAH
5LTY, 6ES2, 6ES3	Q_6_	Helical, SAH
4FE8, 4FEB, 4FEC, 4FED, 4WTH	Q_7_HQHQHQ_27_	Helical, turn, β-hairpin
1QBK	ED_6_EID_4_	Disordered, turn, helical, SAH
2WGO	D_6_	Disordered
5GAP, 5GAN	DIDEVD_6_	Turn, beta-bridge
5GRQ	D_6_ND	Bend, disordered
1AP0, 1GUW	E_6_	Turn, disordered
1L0L, 1NTK	E_8_	Helical, SAH
1MHS	EDDEDEDID, E_6_	Bend, disordered
2MKF, 2MKG, 2RR9	QE_6_	Helical, SAH
2XZE	EDE_6_	Helical, coiled coil with oppositely charged (multiple K) α-helix
6EYC, 3JA8, 5BK4, 3JC5, 3JC7, 5XF8, 5H7I, 5U8S, 5U8T, 6F0L, 6HV9	E_6_	Helical, orthogonal to another helix
5A6C	NE_6_D	Turn
5E26	E_6_	Helical, SAH
5IY6, 5IY7, 5IY8, 5IY9, 5IVW	DKDE_6_	Turn, disordered
5XIS, 5XIT, 5YDK	E_6_	Helical, SAH
5XTE	E_8_	Helical, SAH

In the initial report of (Q/N)-rich and polyQ regions in human proteins forming coiled-coil structures in vitro, the coiled-coil propensities of these regions were predicted with COILS and PairCoil2^56^. However, it has already been reported in 1995 and was described as major intention to develop a new algorithm, that COILS predicts coiled coils in homopolymers of charged amino acids^21^. This did not change with the second and current COILS version, NCOILS. NCOILS predicts coiled coils in homopolymeric polyK, polyE, polyN, and polyQ peptides, and in polyR and polyA peptides if additional amino acids are inserted somewhere in the homopolymer. A single leucine within polyR is enough to turn this homopolymer into a “coiled coil”, and for polyA to become a “coiled coil” two glutamates or a glutamate and a lysine are needed. The latter polyA[+ 2E/EK] is also predicted to be a “coiled coil” by Multicoil. Marcoil also predicts “coiled coils” for homopolymeric polyK, polyE, and polyQ peptides. These predictions can easily be reproduced by the reader using Waggawagga, a webserver for the comparative analysis and visualisation of coiled-coil predictions of the most common coiled-coil prediction software packages^57^. Thus, the prediction of coiled coils for such homopolymers is rather an artefact of these software. Inspection of stretches of at least six consecutive aspartates and glutamates in protein structures supports this conclusion (Table 1). Homopolymers of at least six asparagines are not present in the PDB.

While polyK, polyE, polyQ, and polyN regions might transiently fold into partially α-helical structures, it is extremely unlikely that these cause specific protein interactions or form coiled coils. Even if such regions formed an α-helix, the helix surface would be indistinguishable and would lead to uncontrolled aggregation in all directions. However, organisms with massive homopolymeric regions such as *Dictyostelium discoideum*^58^ do not show more protein aggregation than any other species, which in turn suggests that these homopolymeric regions rather form single α-helices and not aggregates. Therefore, we suggest using the term “coiled coil” exclusively in the original sense coined by Francis Crick for two or more α-helices in a dedicated structural packing, and not for any stretch of amino acids that might partially fold into α-helices and aggregate. In this sense, polyK, polyE, polyQ, and polyN (and polyR and polyA) regions do not form coiled coils.

## Discussion

Coiled coils consist of a minimum building block of just an α-helix and can be designed on a drawing board based on a poly-alanine backbone and subsequently substituting alanines by hydrophobic, charged, and polar amino acids to obtain structures with certain characteristics, mainly a certain length and topology^14,15^. The simplicity in design should, in principle, allow a relatively accurate and precise prediction of these motifs in real-world sequences. For the evaluation of the performance of coiled-coil predictions in the context of a functional genome annotation the reference data set should be large and diverse, should contain only a few percent sequences with coiled-coil regions, and should allow structural verification. To our knowledge the protein structure databank (PDB) represents the most comprehensive reference data set given the broad sampling of species, protein families and protein folds. As ground truth and reference we used the coiled coils detected by SOCKET, which identifies knobs-into-hole packings of α-helices within protein structures. We evaluated the performance of coiled-coil prediction tools against all unique sequences within the PDB using all common binary classification metrics. The specificity and accuracy of all prediction tools is very high, which is a natural result from the large proportion of true negatives within the data set. In contrast, the sensitivity and precision are rather low. This is, in part, due to the lower total numbers of predictions compared to the number of SOCKET hits (however, NCOILS predicted about four times more coiled coils), but more importantly the result of the large proportion of false positive predictions (predicted coiled coils where SOCKET did not find any). Coiled coils were predicted in 31,040 PDB files where no SOCKET hits were found, and thousands of coiled coils were detected within PDB files that do not overlap with SOCKET hits. The structures for which SOCKET might fail explain some dozens of these predicted coiled coils and even a few hundreds, but not the thousands of false positive predictions. It is highly unlikely that SOCKET missed many “classical coiled coils”, which are the supposed primary target of the prediction tools. Without having inspected all predictions manually, we suspect that it is more likely that most of these predictions are in fact false positive hits. Very obvious cases of false positive hits include the prediction of coiled coils in polyQ regions, which are not supported by structural data, and the prediction of coiled-coil regions in sequences that form β-strands, loops and other non-α-helical structures.

The low sensitivity of the prediction tools comes with a high number of false negatives (SOCKET-detected coiled coils that were not predicted). In 3401 PDB files coiled coils were exclusively found by SOCKET. At first instance, the most likely explanation for these cases is that the coiled-coil prediction tools are thought to be specific for solvent-exposed, left-handed coiled-coil dimers, and are not expected to detect types of coiled-coil α-helices buried within globular domains or as part of transmembrane structures. And because most coiled-coil prediction tools were developed before next-generation sequencing boosted sequence databases, the relatively low number of training data for tool development could have also been limiting in detecting more divergent coiled-coil types. However, the false negative rates (1—sensitivity; also called miss rate) of the individual tools at the sequence level are in the range of 63.5% (NCOILS) to 93.4% (PairCoil) indicating that the majority of even the classical coiled coils are not detected. Our analysis also shows that the amino acid patterns at the heptad positions of SOCKET hits overlapping and not overlapping with predictions are very similar. This implies that classical coiled coils and coiled coils within globular structures have similar amino acid distributions suggesting that most of the SOCKET hits in the 3401 PDB files could have been identified by coiled-coil prediction tools just as well as those that were detected.

The discussed metrics depend on the proportion of true and false positives and negatives in the benchmark data set. As discussed, if the fraction of true positives (coiled coils) is low compared to true negatives (no coiled coil), specificity and accuracy will automatically be high, if only 50% of the predictions are correct (Fig. 1). If the fraction of true positives is high compared to true negatives, the sensitivity will automatically be high. Fortunately, there is a metric termed the “Matthews correlation coefficient” (MCC) that is insensitive to the proportion of true positives (coiled coils) in the data set and that gives a balanced assessment of the performance^50^. According to this metric, the performance of the coiled-coil prediction tools at the PDB file level was rather poor (MCCs between − 0.05 and 0.19 when requiring only the overlap of a single amino acid between SOCKET hits and coiled-coil predictions) and did not significantly change at the sequence level (MCCs between 0.02 and 0.22). The MCCs do not considerably increase if the 3401 PDB files with exclusive SOCKET hits are regarded as true negatives (no coiled coils to be detected by prediction tools) and if the 230 PDB files with coiled coils predicted by all tools but not detected by SOCKET are regarded as true positives. Requiring only a single amino acid overlap is a rather weak criterion and balances possible biasing effects from software parameters such as the SOCKET packing-cutoff and window sizes or cutoffs from prediction tools. When requiring a more realistic overlap of at least 50% of predictions and SOCKET hits the quality of the prediction tools is no better than random, based on the MCCs (Supplementary Fig. S8). Independent of whether comparisons and analyses of coiled-coil prediction tools report high sensitivities, specificities, accuracies, and precisions, this analysis of the entire PDB using the MCC as a balanced measure demonstrates that it is random whether a predicted coiled coil in an unknown sequence is a coiled coil or not. In fact, the performance of the tools is very similar to a naïve model assuming that the prediction is random but knows and reproduces the proportion of the reference category in the data set.

The finding that coiled-coil prediction tools show low performance when benchmarked with sequences from heterogeneous protein structures is not completely new. A comparison of SpiriCoil, Marcoil, and PairCoil2 revealed a similar low absolute performance, with SpiriCoil, the supposed best performing coiled-coil prediction tool in this comparison, displaying a sensitivity of 41.7% and an FDR of 84.6% at the level of sequences^32^. In this comparison, 2.7% of the sequences in the test data contained coiled coils, which is slightly lower than the percentage of likely coiled-coil regions in our data set (7.4% of 144,270 PDB files contained SOCKET hits). However, SpiriCoil just passes the coiled-coil assignment from SUPERFAMILY protein profiles on query sequences based on global sequence comparisons without ever verifying the presence of a coiled coil. This approach therefore ignores domain gain, loss, and rearrangement processes, which are very common in eukaryotic genomes. In contrast, the latest comparison showing good sensitivity and specificity of the prediction tools was based on a highly biased sequence data set with 63.4% of the 1643 test sequences containing coiled coils, and each of these sequences containing 2.09 coiled-coil regions on average^39^. Already an even and random assignment of coiled coils to sequences of this data set would result in sensitivity and specificity of 79% and 100%, respectively. Because of the biased benchmark dataset, the implied quality of the coiled-coil prediction tools based on the excellent values for the metrics is completely misleading. In addition to these general shortcomings in approach and data set, in both previous studies the precise location of the coiled coils with respect to reference SOCKET hits and secondary structural elements was not determined.

Given this analysis and the application of the evaluated prediction tools, especially NCOILS, in the functional annotation of genomes it is highly questionable that many of the proteins with predicted regions really contain coiled-coil domains. In addition it is highly likely that coiled-coil domains have been missed in many proteins. Given the broad application of coiled-coil prediction tools, as citation rates suggest, and the high interest in this structural motif, as publication numbers suggest, we see a high demand for accurate coiled-coil prediction. We suggest improving the tools’ performance against unbiased and not pre-selected data and to use approaches that combine sequence profiles and secondary structure assignments, or that discriminate against certain atypical features. Secondary structure information, for example, was included in WDSP, a pipeline to predict WD40 repeats and domains^59^. WD40 repeats have very low sequence homology, are therefore notoriously difficult to detect, and are usually present in a chain of seven repeats folding into a domain^60,61^. In WDSP, protein sequences are filtered by selecting fragments with β-sheets according to PSIPRED^62^. Subsequently, WD40 repeats are detected using a profile generated by aligning repeats by secondary structure elements and not global similarity, and finally WD40 domains are assigned when chains of at least six WD40 repeats are present. As another example, Waggawagga uses the discriminative approach to detect stable single α-helices (SAH domains), which coiled-coil prediction tools mis-predict as coiled coils^57,63,64^. Waggawagga searches for networks of oppositely charged residues and discriminates against helix-breaking residues, networks of residues with identical charge, and networks of hydrophobic residues as found in the hydrophobic seams of coiled coils. Approaches similar to those used by WDSP and Waggawagga could be implemented to improve coiled-coil predictions. For example, protein sequence regions could be pre-filtered and/or coiled-coil predictions could be post-filtered by secondary structure predictions. A selection filter for potentially coiled-coil domain containing regions could also be the detection by at least two tools. Training the prediction tools against unbiased data such as the entire PDB could also improve tools’ performance. The evaluation of multiple tools to predict the pathogenicity of SNPs^65^ and protein stability^66^ also demonstrated low performance (low to medium MCCs), but the results stimulated substantial tool improvement with respect to the benchmark data sets.

In conclusion, at best, the evaluated tools predict coiled-coil regions in well described and well analysed coiled-coil forming proteins with reasonable accuracy. For predicting coiled coils in large data sets with balanced proportion of all protein folds, such as present in gene prediction datasets, the tested tools have only limited applicability. One possibility to reduce the number of false predictions in such functional genome annotations would be to only accept coiled-coils regions if predicted by multiple prediction tools and to only predict coiled coils in regions not already covered by other protein domain predictions.

## Methods

### Benchmark dataset

To benchmark the performance of coiled-coil prediction software as fairly and reliably as possible, we created a copy set of the current state (15/12/2018) of all available 147,073 PDB structures from the RCSB Protein Data Bank^67^. The flatfiles were downloaded, stored locally and parsed with BioRuby v.1.5.1^68^. Removing nucleotide-only structures and some PDB-files with handling issues reduced the number of usable structures to 144,270. Main reasons for handling issues were unavailability (moved/renamed/discontinued) at the RCSB servers (761 files), and BioRuby parsing issues with some of the structures in the PDBx format, some early structures from last century, and structures with non-natural amino-acids. We refrained from any attempt to manually remove PDB files, which could be part of the training data of some of the tools. All six tools benchmarked used mainly collections which are known to contain coiled-coil sequences, such as intermediate filament proteins, muscle myosins, kinesins, tropomyosins, dyneins (for an extended list see^23^), and, if at all, only a few sequences derived from the PDB. Given the number of sequences used in this benchmark here, the effect of the few PDB sequences already present in the training data of some of the tools should be marginal. In any case, the performance of the tools might look slightly better in the benchmark than is in practice. The information from PDB files and all additional data generated were stored in a PostgreSQL database. To facilitate data handling and analysis of the PDB, DSSP, SOCKET, and coiled-coil prediction information a relational database scheme was designed, which stores the relevant data for the evaluation with low redundancy and depicts each data type into its assigned classes (Supplementary Fig. S10). Protein sequences were extracted from the ATOM records of the PDB files. Identical sequences (from start to end including identity of possible gaps) were removed independent of whether they were present in the same or different PDB files. This means that sequences differing by a single amino acid at, for example, the N- or C-terminus because of their presence in different structures or independent molecules within the asymmetric unit are treated as different sequences. The remaining sequences break down to 187,776 unique sequences. In order to create a broad, representative data set, these unique sequences remained unreduced in terms of similarity or other criteria, even very short sequences were left in the data set. Only the 755 sequences containing amino acids labeled “unknown”, one-letter code “X”, which are handled very differently by the coiled-coil prediction tools, were removed from the analysis. The remaining 187,021 sequences were used as reference for all analyses. Accordingly, the secondary structure for every single amino acid within the reference sequences is known. The overall size of the database for the current PDB structure data set amounts to 2.9 GB, the flatfiles in combination with the predictions sum up to around 159 GB in 2.8 million files.

### Running coiled-coil software

The SOCKET algorithm detects knobs-into-holes packed α-helices based on secondary structure assignments from DSSP v.2.0.4 (Define Secondary Structure of Proteins)^54,55^, which we generated according to the software documentation. DSSP only needs specification of an input and an output file. For the determination of coiled-coil regions SOCKET^37^ was run with the recommended parameter settings, especially the packing-cutoff was left at the default 7 Å as described in the documentation. The coiled coils were, according to the database model, split into their superordinate structure and building/participating components, which contain registers, sequences and position information. To prevent mis-assignment of any amino acid due to sequence gaps or other unexpected shifts, the register assigned sequence of each SOCKET-determined coiled-coil component is searched in the respective PDB sequence and a potential offset is added to the component database entry.

Each coiled-coil prediction software was run with its recommended default settings and a search window of 21 amino acids, except for Marcoil and MultiCoil2, which are implemented to run without a respective window setting. Accordingly, quite conservative thresholds were set for coiled-coil selections. For Marcoil a lower limit of 90.0 (minimum) was chosen (HMM training file 9FAM, with default transition and emission parameters). For MultiCoil and MultiCoil2, the “CoiledCoil-Threshold” was set to 0.25 (minimum). For NCOILS, the “CoiledCoil-Threshold” was 0.5 (minimum), and the latest provided MTIDK-matrix was used. For PairCoil and PairCoil2, the “CoiledCoil-Threshold” was 0.84 (minimum) and 0.025 (maximum), respectively. Because the reference sequences do not contain the gap information as present in protein structures, coiled-coil prediction tools handle all sequences as continuous entities. This might affect the length of some predicted coiled coils.

### Data reduction

For generating data sets with reduced sequence redundancy, the PostgreSQL database was copied three times and the unique sequences were subjected to CD-hit^49^ applying 90%, 70% and 50% sequence identity cut-offs, respectively. Each of the now four databases were copied another two times and region length cut-offs of 14 and 21 amino acids were applied to the stored reference SOCKET regions and the coiled-coil predictions.

### Determining overlap between assignments and predictions

Assigning SOCKET hits, DSSP assignments, and coiled-coil predictions globally to PDB files and chains is trivial. However, precisely determining overlapping regions within sequences is more challenging because amino acid numbering schemes change with data parsing and gaps in structures. Numbering of amino acids in DSSP features and SOCKET hits follows the numbering of the amino acids in the structures, which either start with the first amino acid of the protein construct, the first amino acid of the sequence of interest (excluding any terminal amino acids from protein expression plasmids), or follow the numbering of the analysed protein with respect to the numbering in the gene or transcript. The numbering of amino acids in the structures is usually aware of gaps. When extracting sequences from PDB files all position-wise numbering information including gaps is discarded, only start- and end-positions in PDB numbering are retained. Sequences themselves are stored plain without any numbering in the database, meaning a sequential numbering starting with “1” when referred to from other tools. Instead of fitting the results from the prediction tools to the complex numbering in the structure files, the numbering of the initial register sequences of the SOCKET hits was shifted to the position-independent numbering as described. Accordingly, the matching between SOCKET hits and coiled-coil predictions is independent of any peculiarities in structure numbering and independent of any gaps in the structures. Similarly, the sequences corresponding to every contiguous DSSP feature in a structure are located in the number-less sequences and the DSSP features are subsequently numbered according to the matching. A problem with this approach could be that very short DSSP features (1–3 amino acids), which are surrounded by sequence without feature (“loop”), might match to more than one position. However, these very short DSSP features are very likely not part of SOCKET hits or coiled-coil predictions so that the matching of DSSP and SOCKET/coiled-coil prediction is not affected.

### Handling difficult cases

There are a few cases which cannot be resolved consistently without introducing multiple subcategories, which in turn would considerably detract from the main message without adding additional understanding. One of these problems is handling cases of overlapping SOCKET hits and coiled-coil prediction when one overlaps multiple of the other. In such cases we treated every overlap independently. For example, a long coiled-coil prediction could overlap with a SOCKET hit in its N-terminal half and another SOCKET hit in its C-terminal half. Such cases were treated as two independent overlap instances.

The actual data categories and assigned categories (true and false positives and negatives) for computing the Matthews correlation coefficient at the level of PDB files are clearly defined. It is, however, difficult to define the same categories (true and false positives and negatives) in case of evaluating the cases of overlapping SOCKET hits and coiled-coil predictions. The problem is that multiple hits were found in many PDB files, and those hits can be overlapping and non-overlapping. As a rough approximation for an upper bound we computed for each tool the percentage of overlapping hits within PDB files, and applied this percentage onto the number of overlapping PDB files. With this approach we rather overestimate the number of true overlapping hits, because for most tools there are more non-overlapping than overlapping hits in PDB files with both SOCKET hits and coiled-coil predictions, and the false positive PDB files contain, to some extent, multiple coiled-coil predictions whose contribution is ignored.

### Viewer for coiled coils mapped to PDB structures

For visual inspection of SOCKET hits and coiled-coil predictions, a simplified search interface to the analysis database and a 3D molecule viewer were integrated into the coiled-coil project site Waggawagga. Structures can be searched by PDB ID and results are displayed for each structure on a single page. The result page presents the structure in JSmol, a JavaScript—only version of Jmol^69^, for interactive viewing, some general information about the PDB file, and all SOCKET hits and coiled-coil predictions if found. The SOCKET hits and coiled-coil predictions are shown in the sequence-based and interactive Waggawagga format^57^ providing access to all information down to the amino acid level. SOCKET hits and coiled-coil predictions can be loaded and combined into the JSmol viewer by simple selection. Intersecting regions between SOCKET hits and predictions are marked separately allowing the structure-based visual inspection of coiled-coil assignment by SOCKET versus prediction tools.

### Handling of multimers not present in the PDB files

Having finished the entire benchmarking study we were made aware by a reviewer comment that we might have missed a substantial part of coiled coils because we did not include multimers, which are not present in the PDB files. PDB files contain the coordinates of the molecules present in the asymmetric units. In few cases, a biologically relevant multimer might be present in the crystal structure, of which only a monomer is present in the asymmetric unit. These multimers can be reconstructed using the transformation matrices present in the BIOMT part of the PDB files. To reconstruct all possible multimers we used the tool MakeMultimer.py (http://watcut.uwaterloo.ca/tools/makemultimer/index). 7180 PDB files were missing BIOMT information. 760 PDB files generated a “__main__.PdbError: invalid pdb code “ error, 675 generated a “list index out of range” error, and 10 generated key or value errors. Subsequently, DSSP and SOCKET were run on all multimers. DSSP failed on 2081 of the generated multimer PDB files. 875 PDB files were obtained that contain coiled coils that were not present in the initial data set. Of these, 9 are not based on multimers but the result of various bugs in the SOCKET output that we were not aware of when checking the SOCKET output files of the benchmark data. In contrast, 487 coiled coils are only present in the initial dataset and not in the makeMultimer.py generated dataset due to the missing BIOMT data and the error messages described above. The total number of missed coiled coils is small compared to the number of coiled coils in the benchmark data, and the coiled coils likely distribute on true positives and false negatives similar to the benchmark data. A few false positives might turn to true positives, but most of the missed coiled soils (SOCKET) will add to the false negatives (no coiled coil predicted), thus numbers of the predictions will mainly shift from true negatives to false negatives. For an example see Supplementary Fig. S11. Given the low number of PDB files with coiled coils, which were predicted by all tools but are not present in the SOCKET reference dataset (see Venn diagram in Fig. 1C, only presence in same PDB file was tested but not overlap) the missed coiled coils will show a similar distribution for true positives and false negatives for each prediction tool as show the coiled coils in the analysed data set.

## Supplementary Information

Supplementary Information 1.

Supplementary Information 2.

Supplementary Information 3.

Supplementary Information 4.

Supplementary Information 5.

Supplementary Information 6.

## Data Availability

The data are freely available at figshare https://doi.org/10.6084/m9.figshare.9994706.
