# Supplementary material for: Critical assessment of coiled-coil predictions based on protein structure data
Source: Sci Rep. 2021 Jun 14;11:12439. doi: 10.1038/s41598-021-91886-w (PMC8203680; doi:10.1038/s41598-021-91886-w)
Supplement: Supplementary file 1 — Supplementary Information 1. [file 41598_2021_91886_MOESM1_ESM.pdf]

Supplementary Material

Critical assessment of coiled-coil predictions based on protein structure data

Dominic Simm[1,2], Klas Hatje[1#], Stephan Waack[2] and Martin Kollmar[1*]

[1] Group Systems Biology of Motor Proteins, Department of NMR-based Structural Biology, Max-Planck-Institute for Biophysical Chemistry, Göttingen, Germany

[2] Theoretical Computer Science and Algorithmic Methods, Institute of Computer Science, Georg-August-University Göttingen, Göttingen, Germany

[*] Corresponding author

E-mail: mako@nmr.mpibpc.mpg.de (MK)

[#] Current address: Roche Pharmaceutical Research and Early Development, Pharmaceutical Sciences, Roche Innovation Center Basel, F. Hoffmann-La Roche Ltd., Basel, Switzerland

Supplementary Notes and References

Limits of previous evaluations of coiled-coil prediction tools

Earlier assessments of coiled-coil tool performance used user-defined subsets of the PDB. However, instead of mining the PDB directly, another database was used, the SCOP Structural Classification of Proteins database,[1] to extract superfamilies with coiled-coil domains for the positives and superfamilies of the alpha and beta protein class for the negatives.[2] Both categories were enriched by homology searches in the nonredundant database followed by sequence alignment and redundancy reduction to result in a final data set of about 1% positives, a highly imbalanced data set. This approach generated hardly verifiable reference data as it involved manual removal of SCOP coiled-coil superfamilies from the positives, which according to SOCKET did not contain coiled-coils, manual removal of SCOP superfamilies from the negatives, which obviously contained coiled-coils, and the sequence search and alignment of the notoriously difficult to align coiled-coil domains. The performance of coiled-coil prediction tools was assessed at the residue level by sensitivity and specificity, which were termed coverage and reliability for unknown reasons.[2]

In a subsequent analysis, all PDB structures deposited in an 18-month period were downloaded, filtered to 95% sequence identity, and split into positives and negatives using SOCKET hits as criterion.[3] With about 2.6% positives this data set was also quite imbalanced. Coiled-coils were evaluated at the protein sequence level, albeit only by counting hits on the same sequence without verifying the precise location of reference and prediction (e.g. overlap was not confirmed).

A later comparative study combined both approaches:[4] The positives were determined by mining the PDB with SOCKET. The negatives were compiled in a difficult to follow procedure of selecting alpha and beta classes from SCOPe, removing classes that contain annotated coiled coils, removing all available training data from prediction tools, recombining the resulting set again with the training data of three tools, and finally applying a 30% sequence identity cut-off using CD-HIT. The final data set contained 1643 sequences, of which 601 did not contain any coiled coil (negatives) and of which 1042 contained 2176 coiled coils (positives). Performance was evaluated as sensitivity and false positive rate (1 – specificity) at the sequence level, e.g. a SOCKET hit and a coiled-coil prediction in the same protein sequence were regarded as true positive hit. It is obvious, that this data set is highly imbalanced towards positives (63%), and that chances to obtain positive hits are additionally

increased by the fact that most of the sequences of the positive class contain multiple coiled coils.

Most recently, test data were compiled by selecting the PDB data clustered by 50% sequence identity, running SOCKET and preferentially selecting structures with coiled coils as representatives of the clusters.[5] Further redundancy in the selected structures was removed by applying CD-HIT to 50% sequence identity. The data were further filtered by structure resolution and minimum and maximum protein sequence length cut-offs. To increase the proportion of the positives, half of the negatives (sequences without coiled coil) were randomly removed. The prediction tools' performance was evaluated using multiple binary classification metrics (except the MCC) at the sequence and residue level.

References

1. Andreeva, A. *et al.* SCOP database in 2004: refinements integrate structure and sequence family data. *Nucleic Acids Res.* **32**, D226-229 (2004).
2. Gruber, M., Söding, J. & Lupas, A. N. Comparative analysis of coiled-coil prediction methods. *J. Struct. Biol.* **155**, 140–145 (2006).
3. Rackham, O. J. L. *et al.* The evolution and structure prediction of coiled coils across all genomes. *J. Mol. Biol.* **403**, 480–493 (2010).
4. Li, C. *et al.* Critical evaluation of in silico methods for prediction of coiled-coil domains in proteins. *Brief. Bioinform.* **17**, 270–282 (2016).
5. Ludwiczak, J., Winski, A., Szczepaniak, K., Alva, V. & Dunin-Horkawicz, S. DeepCoil-a fast and accurate prediction of coiled-coil domains in protein sequences. *Bioinformatics* **35**, 2790–2795 (2019).
6. Simm, D., Hatje, K. & Kollmar, M. Waggawagga: comparative visualization of coiled-coil predictions and detection of stable single α-helices (SAH domains). *Bioinformatics* **31**, 767–769 (2015).

Supplementary Figures

1A

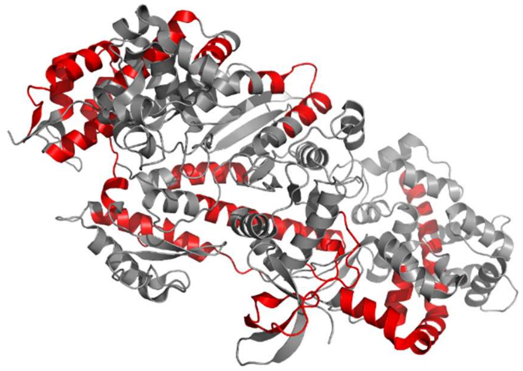

1B

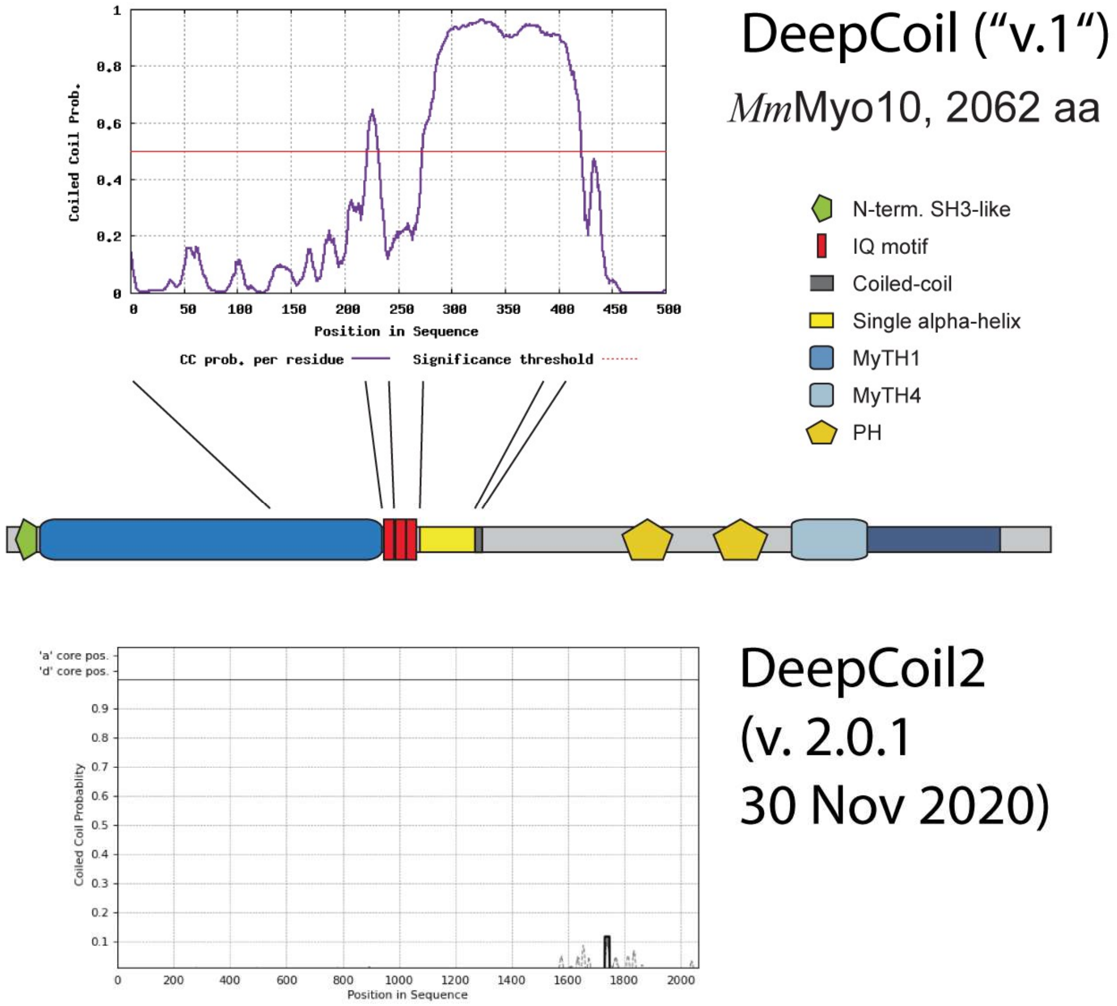

Supplementary Figure S1. Prediction of coiled-coils in human MyoVI with CCHMM-PROF, and in mouse MyoX with DeepCoil and DeepCoil2.

A) To test the performance of CCHMM-PROF, which is only accessible via a web interface, the motor domain sequence of human MyoVI was used. MyoVI is a backwards walking myosin motor, and was chosen by chance from the list of available protein crystal structures of myosin motor domains. Predicted coiled-coil regions were mapped onto the crystal structure, PDB ID 2BKH, and are shown in red. B) To test the performance of DeepCoil and DeepCoil2, which are accessible via a web interface, the mouse MyoX was used. The output of DeepCoil was mapped onto the domain architecture for better orientation. Surprisingly, while there is coiled-coil probablility for a large region from DeepCoil prediction, DeepCoil2 does not predict any coiled-coil region.

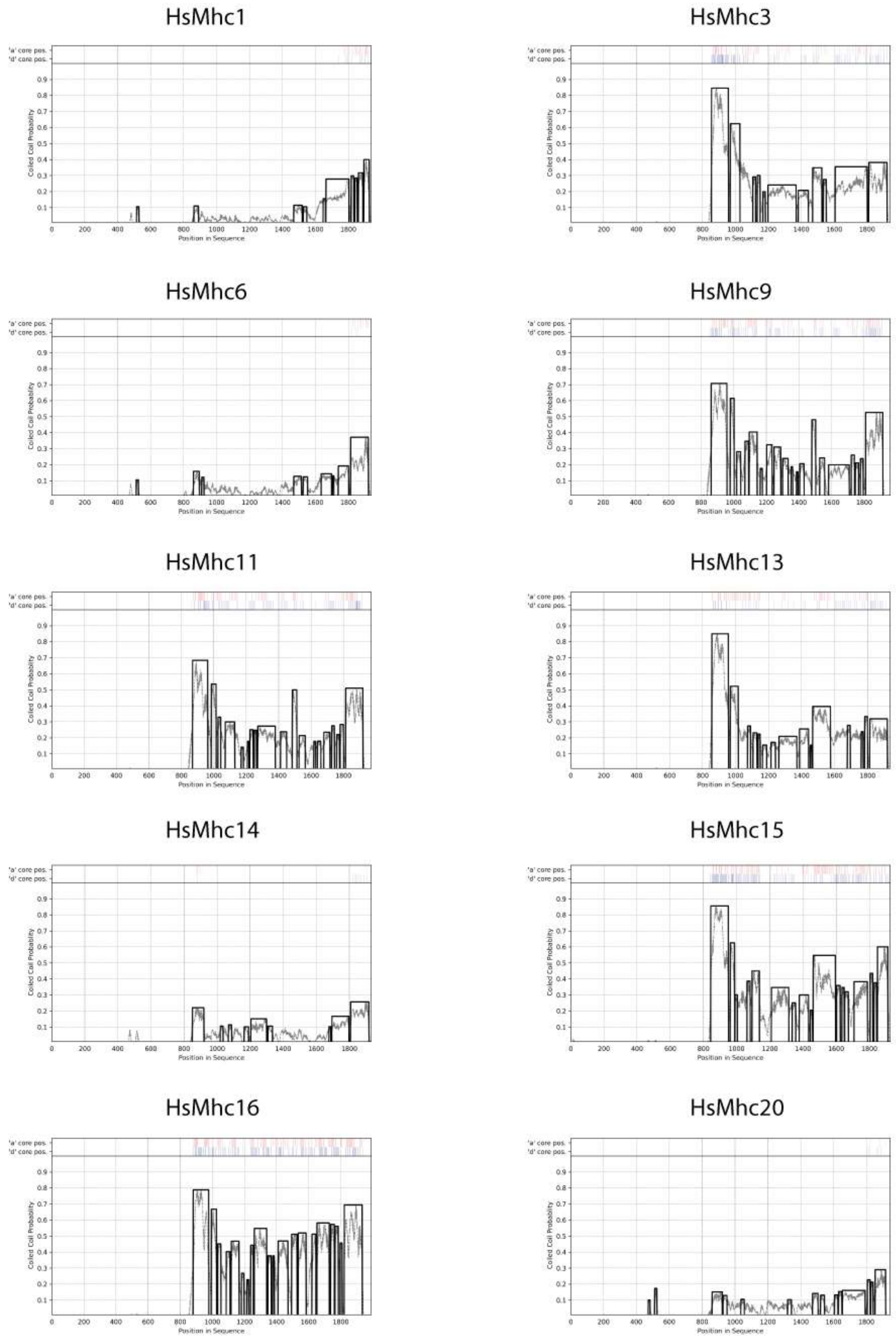

Supplementary Figure S2. Prediction of coiled-coils in human muscle and non-muscle myosin heavy chain proteins (class-2 myosins) using DeepCoil2 (v. 2.0.1. 30 Nov 2020).

To test the performance of DeepCoil2 we predicted coiled-coil regions in several of the classic coiled-coil forming proteins, the muscle and non-mucle myosin heavy chain proteins from human. For protein naming please check Mühlhausen & Kollmar, BMC Evol Biol 2017. Predictions by the tools analysed in this benchmark study are shown in figure S5.

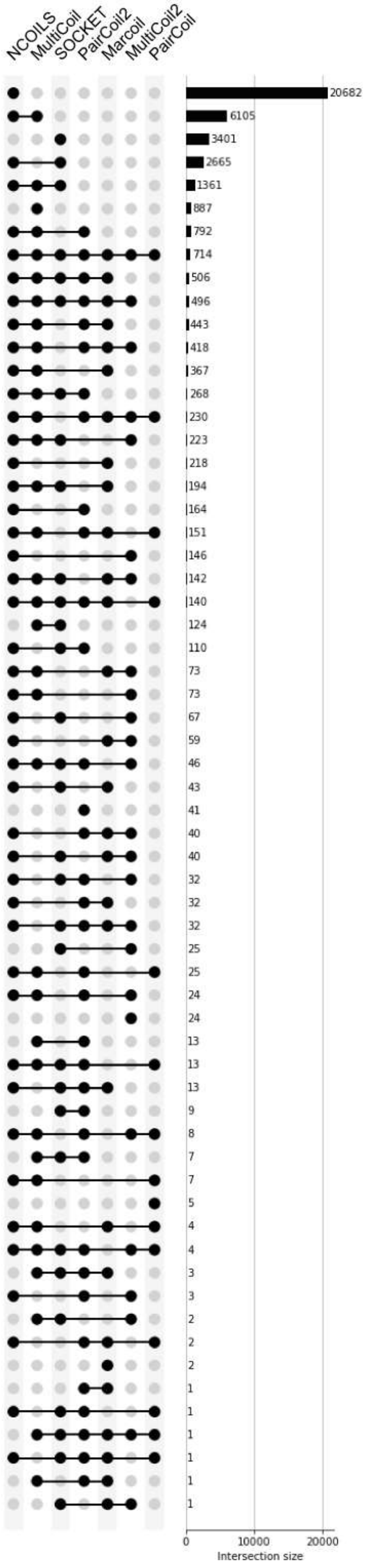

Supplementary Figure S3. Upset plot of the intersection of SOCKET hits and coiled-coil predictions.

The upset plot is based on the same data presented in main Fig 1C.

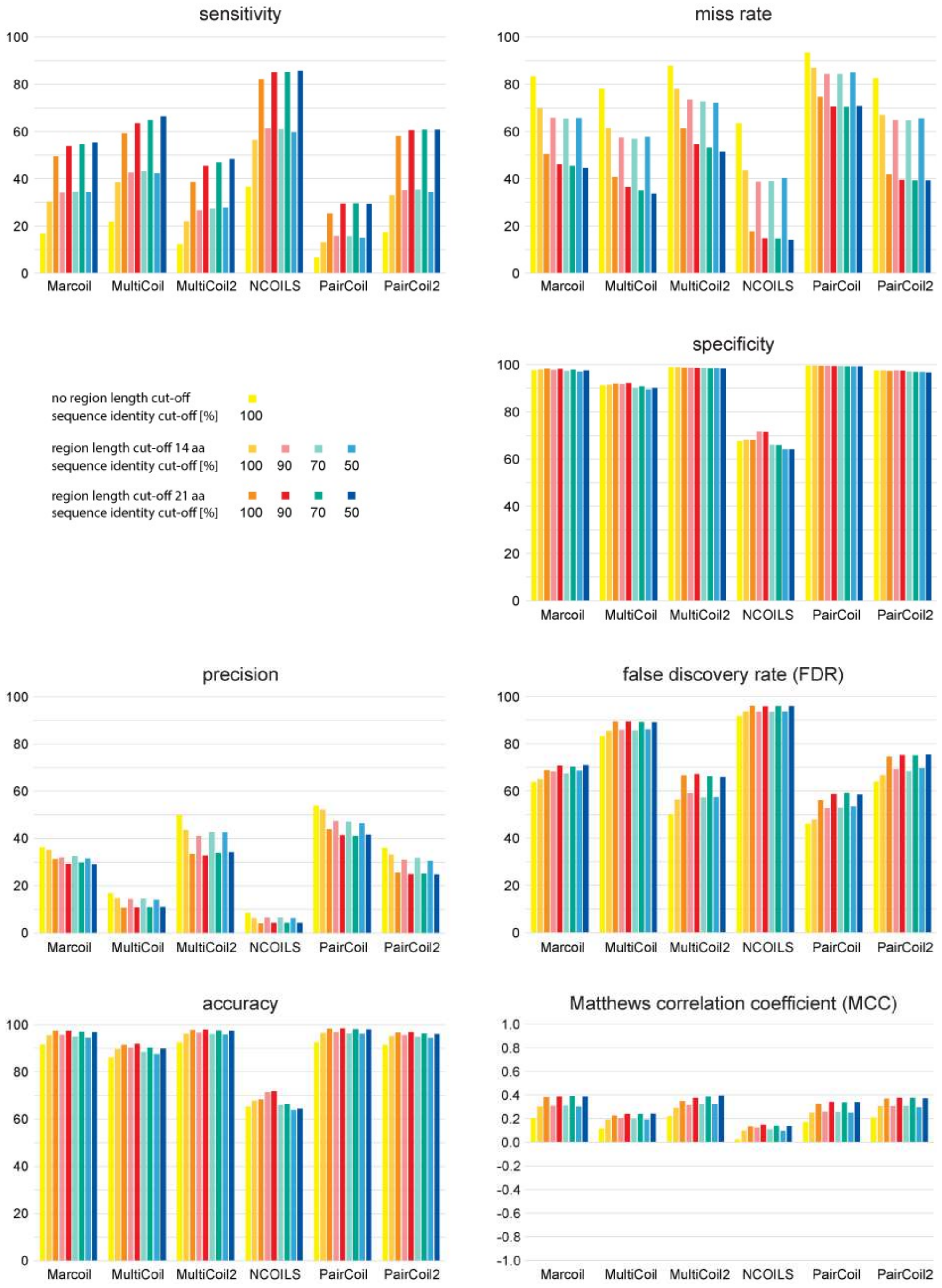

Supplementary Figure S4. Performance of coiled-coil prediction tools in dependence of reference sequence length.

Several classification metrics are shown for the six coiled-coil prediction tools with respect to SOCKET coiled-coil identifications. Classification as true positive hit requires overlap of at least a single amino acid between prediction and SOCKET. The performance was analysed for data sets with increasing minimum length of coiled-coil region (SOCKET hit or prediction) and decreasing levels of sequence redundancy.

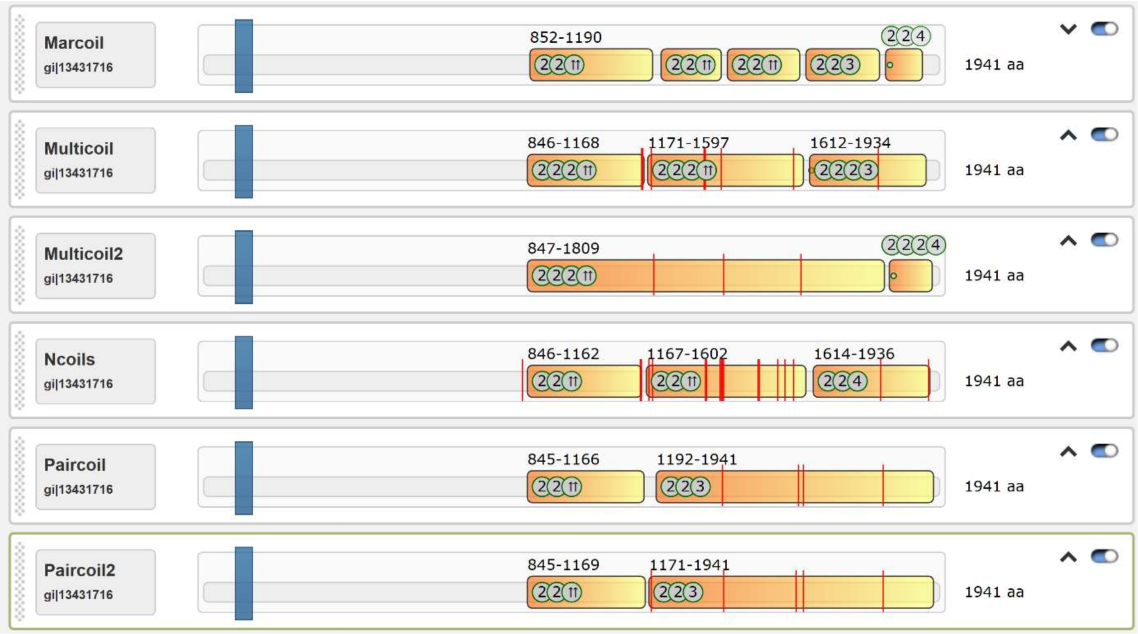

Supplementary Figure S5. Coiled-coil prediction on human adult skeletal myosin heavy chain protein. The coiled-coil tools of interest were run on the human myosin heavy chain 2a protein sequence (skeletal muscle, adult 2 variant; GenBank identifier 13431716). The orange bars denote the predicted coiled-coil regions with their range given in numbers on top of the bars. Red lines indicate interruptions of heptads, regular breaks such as stutters and stammers as well as any other breaks. The grey circles show the predictions of oligomeric states with from left to right: MultiCoil or MultiCoil2 (only for the MultiCoil predictions), SCORER 2.0, PrOCoil and LOGICOIL (LOGICOIL also predicts parallel or anti-parallel dimeric coiled coils, which are indicated by arrows here). The blue bars are sequence sliders for detailed helical wheel and helical net views and can be ignored here. The image has been generated with the Waggawagga tool.[6]

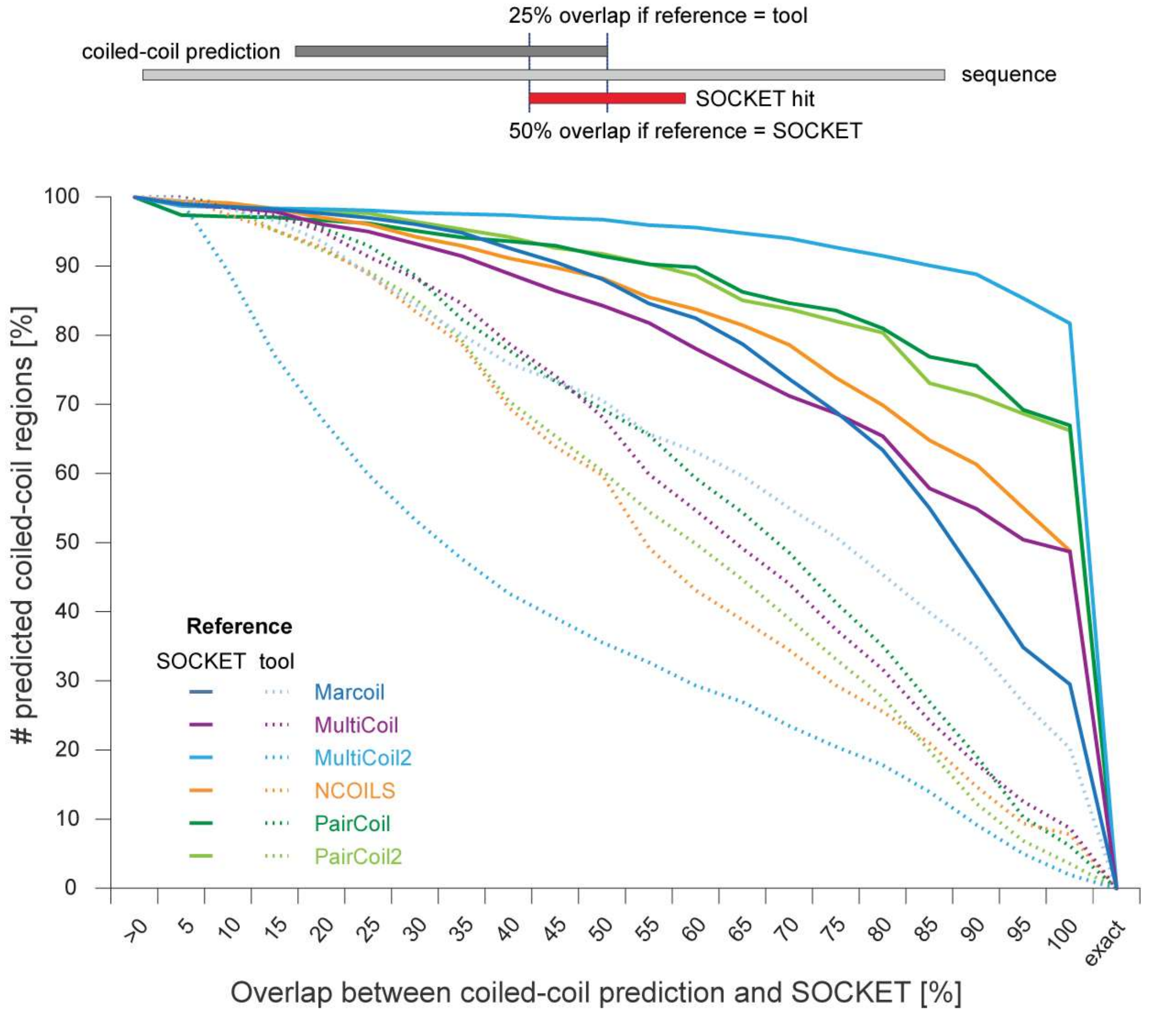

Supplementary Figure S6. Overlap of coiled-coil predictions with SOCKET hit regions. Top: Schematic drawing of a coiled-coil prediction overlapping a SOCKET hit. The ratio of overlap between prediction and SOCKET hit is different depending on whether the prediction or the SOCKET hit is taken as reference.

Bottom: The plot shows the percentage of coiled-coil predictions in dependence of the degree of overlap with the SOCKET hits. For each tool, the percentage of overlapping hits is counted once with taking the coiled-coil prediction as reference (solid lines) and once with the SOCKET hits as reference (dashed lines). The percentage of PDB files with predictions overlapping a SOCKET hit with at least a single amino acid was set to 100%.

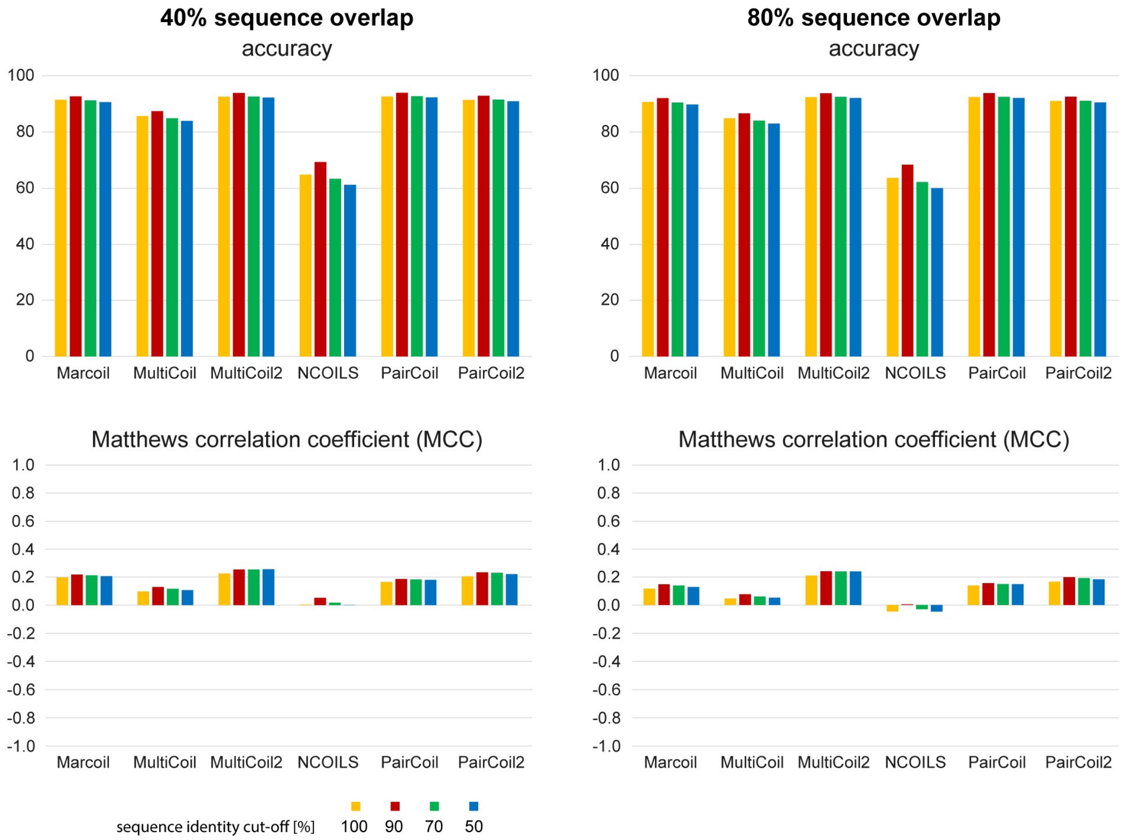

Supplementary Figure S7. Performance of coiled-coil prediction tools in dependence of overlap with SOCKET reference. Accuracy and MCC are shown for the six coiled-coil prediction tools with respect to SOCKET coiled-coil identifications. Classification as true positive hit requires 40% (left plots) and 80% (right plots) overlap between SOCKET and prediction. Percentage overlap was determined with respect to the SOCKET hit.

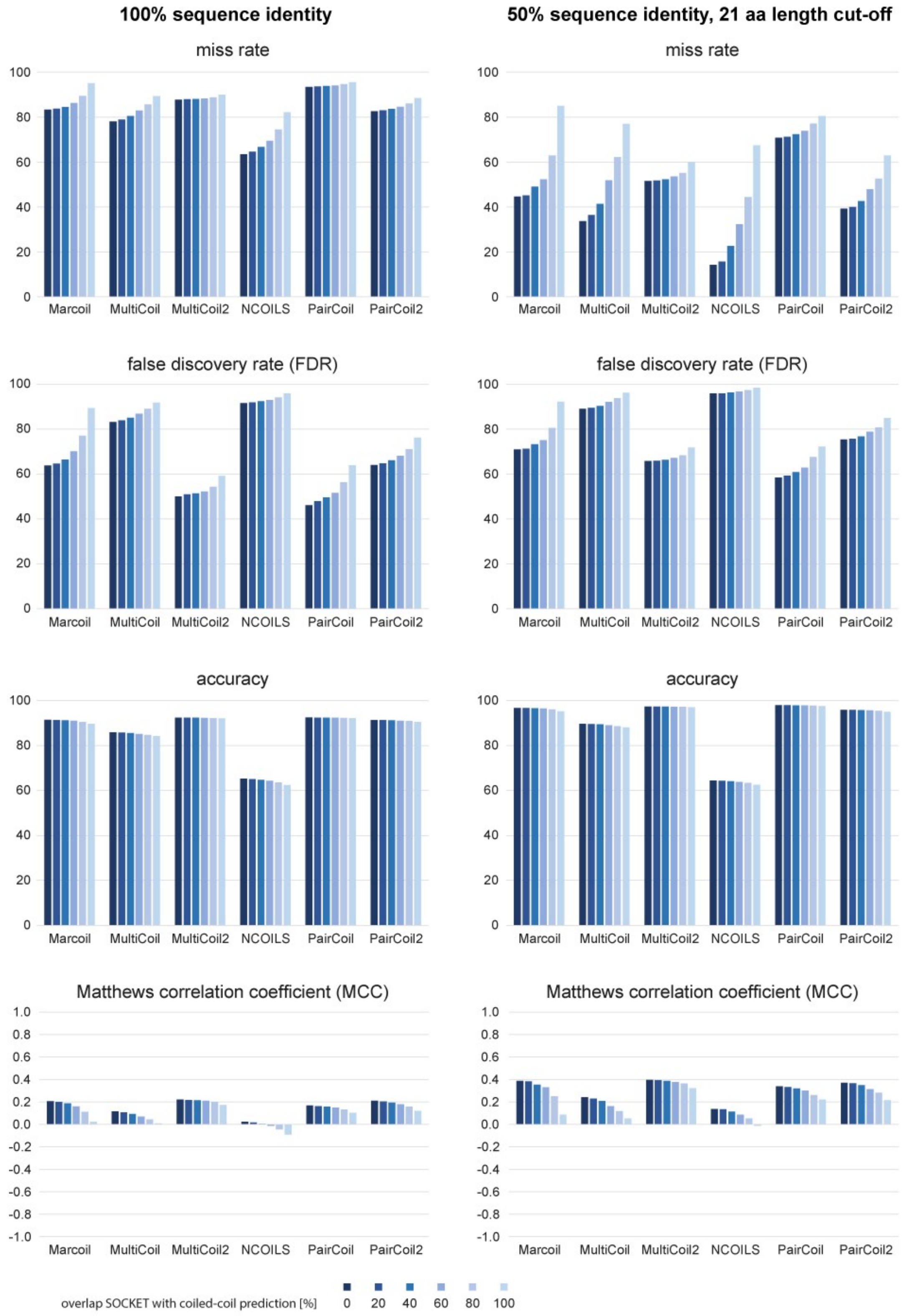

Supplementary Figure S8. Performance of coiled-coil prediction tools in dependence of overlap with SOCKET reference. Several classification metrics are shown for six coiled-coil prediction tools with respect to SOCKET coiled-coil identifications. Classification as true

positive hit requires overlap between prediction and SOCKET. The performance was analysed for two data sets. Plots on the left column: no filter. Plots on the right column: minimum length of 21 amino acid for coiled-coil regions and a sequence redundancy cut-off of 50%. For each data set, the metrics were computed for increasing percentages of overlap between prediction and reference. Percentage overlap was determined with respect to the SOCKET hit.

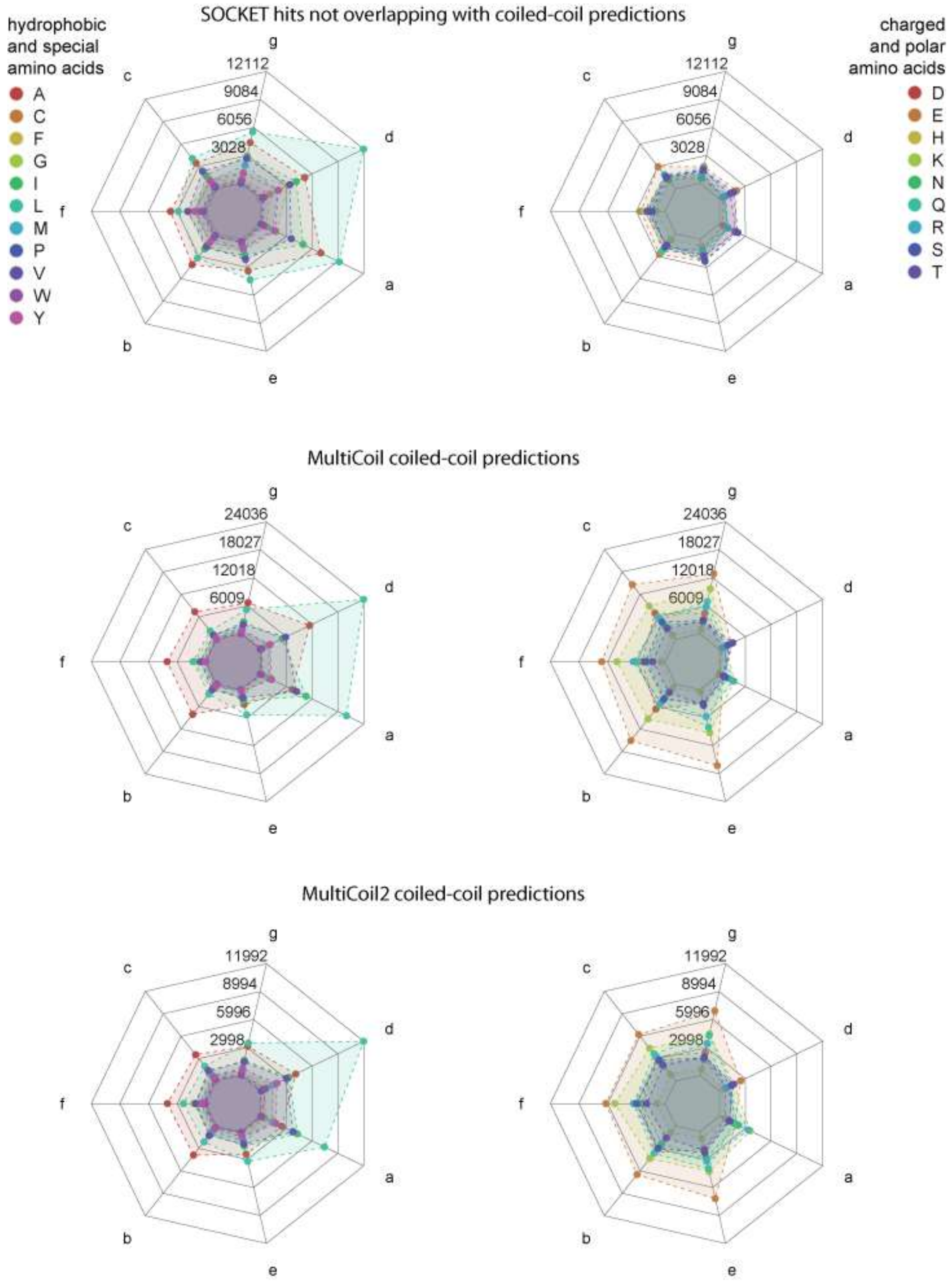
SOCKET hits not overlapping with coiled-coil predictions
hydrophobic and special amino acids
A
C
F
G
I
L
M
P
V
W
Y
charged and polar amino acids
D
E
H
K
N
Q
R
S
T
g
d
a
e
b
f
c
12112
9084
6056
3028
MultiCoil coiled-coil predictions
24036
18027
12018
6009
MultiCoil2 coiled-coil predictions
11992
8994
5996
2998

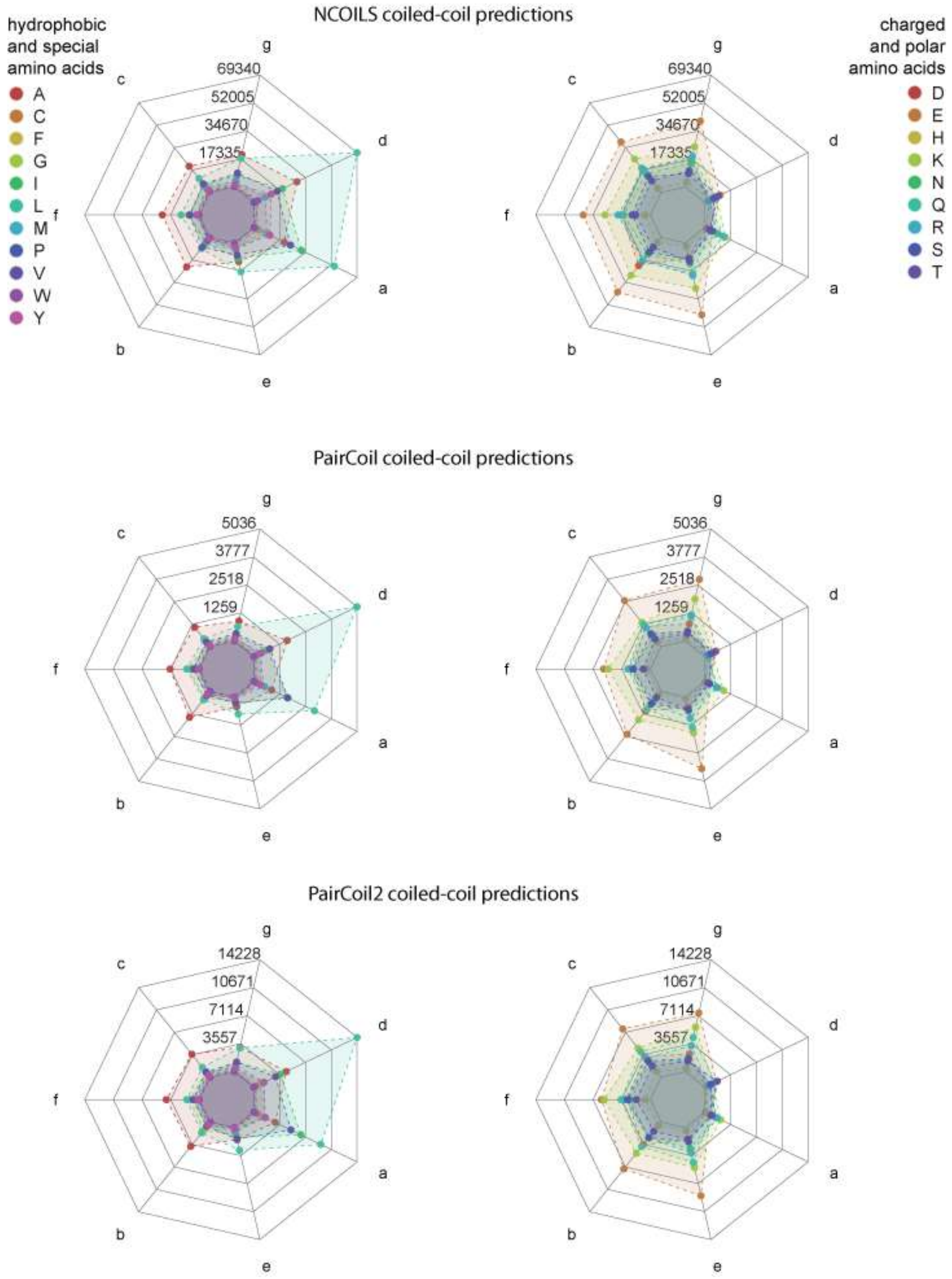

Supplementary Figure S9. Amino acid preferences at heptad positions *abcdefg*.

It is well known that hydrophobic amino acids are preferred at the interface between coiled α-helices, at positions *a* and *d*, and that charged and polar amino acids are preferred at the outside, especially at positions *e* and *g*. Because SOCKET not only detects "classical" coiled-coils but interacting α-helices within globular protein structures, the distribution of hydrophobic and charged amino acids is slightly less biased in the latter structures. The heptad patterns of the coiled-coils predictions show strong bias for leucine and isoleucine at the interior positions *a* and *d*, and for glutamate at all other positions, independently of the

tool. The letters at the axes denote the heptad register positions. Data values at grid lines refer to amino acid counts at each heptad position over all heptads.

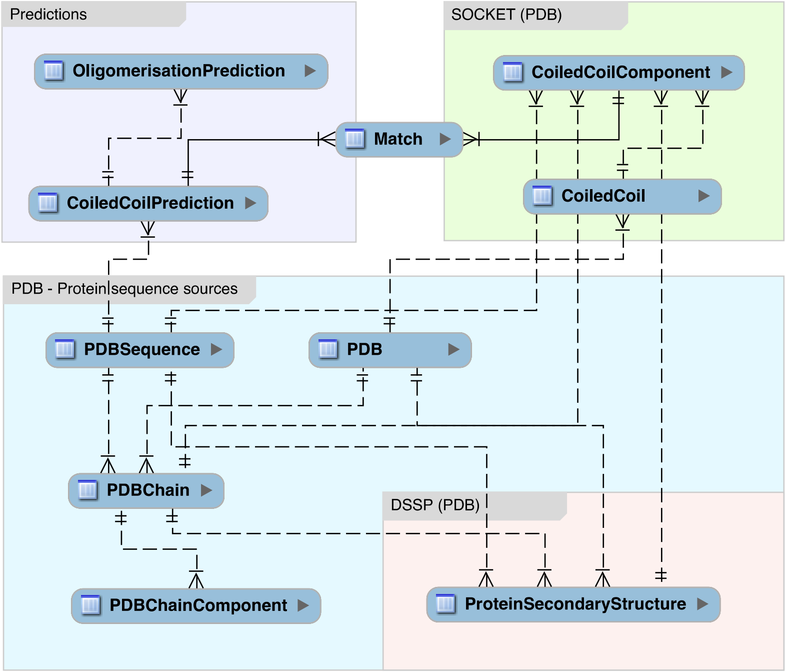

Supplementary Figure S10. Database scheme.

Only the table names and connections are shown for clarity.

A. 4AU6, 3ZRY, 5J7V, 4N5C, 2R17, 4B2Q, 5HIU, 2WPD, 6B2Z, 6B8H, 6BA1, 6FF6, 4CFE, 4CBJ, 6AP4, 2XOK, 1I5N

B. Incomplete output

```
assigning heptad to helix 17 (Y) 60-77:D
extent of coiled coil packing:  63- 77:D
sequence TILQETTHLXENLLDEAR
register    abcdefgabcdefga
partner  --!--!X---X--X----
knobtype --2--44---4--4----
repeats   0 non-canonical interrupts in  15 residues: 7,7,1


coiled coil 15:
        angle between helices  0 and  1 is  167.145     antiparallel
        angle between helices  0 and  3 is  161.145     antiparallel
        angle between helices  1 and  2 is  162.695     antiparallel
        angle between helices  2 and  1 is  162.695     antiparallel
        angle between helices  2 and  3 is  146.836     antiparallel
        angle between helices  2 and  4 is   23.195     parallel
        angle between helices  3 and  2 is  146.836     antiparallel
        angle between helices  3 and  4 is  149.257     antiparallel
this coiled coil is antiparallel
could not find a complementary knob to knob 23 in daisy chain 0
```

Correct output

```
assigning heptad to helix 2 (Y) 60-77:A
extent of coiled coil packing:  63- 77:A
sequence TILQETTHLXENLLDEAR
register    abcdefgabcdefga
partner  --!--!X---X--X----
knobtype --2--44---4--4----
repeats   0 non-canonical interrupts in  15 residues: 7,7,1
1I5N_mm4.pdb c  7.00 e 0 result 1 COILED COILS PRESENT (+ 2 helix groups are either
pairs with too few complementary kno
b in hole interactions or are subsets of larger coiled coils)
Finished
```

C.

Chain-B	aa 13-23	aa 40-54	aa 62-77	aa 78-87	aa 95-106
SOCKET	Yes	Yes	Yes	No	Yes
MarCoil	FN	FN	FN	FN	FN
MultiCoil	FN	FN	FN	FP	FN
MultiCoil2	FN	FN	FN	FN	FN
NCOILS	FN	FN	single long coiled coil		
PairCoil	FN	FN	FN	FN	FN
PairCoil2	FN	FN	FN	FN	FN

Supplementary Figure S11. SOCKET coiled coils not present in the benchmark data due to SOCKET output problems and missing multimers.

A) PDB files containing SOCKET coiled coils where the output file of the SOCKET run is incomplete. B) Incomplete output of SOCKET run on 1I5N.pdb, and SOCKET run on one of

the generated "multimers" of 1I5N.pdb. C) Example of the performance of the prediction tools on a coiled coil missed in the benchmark data set, chain-B of 1I5N.pdb. 1I5N.pdb contains 4 chains of identical sequence but slightly different structure. For chain-A, a 5-stranded coiled coil is predicted by SOCKET, but the output is incomplete not providing the detected registers. Chain-B is shown in the figure. Chain-C is identical to chain-B. Chain-D is predicted by SOCKET to contain a 2-stranded coiled coil (aa 40-54 and aa 62-77).

S1 Table. Numbers and performance metrics of coiled coils and predictions (PDB-version: 2018-12).

S2 Table. Numbers and performance metrics of coiled coils and predictions with respect to redundancy, including length cut-offs.

S3 Table. Analysis of matches between predictions and all coiled-coil components.

S4 Table. Numbers and performance metrics of coiled coils and predictions with respect to redundancy and various overlap cut-offs.

S5 Table. Analysis of matches between predictions and ProteinSecondaryStructures (DSSP)
